# Use of medicinal plants for headache, and their potential implication in medication-overuse headache: Evidence from a population-based study in Nepal

**DOI:** 10.1177/0333102420970904

**Published:** 2021-01-12

**Authors:** Elise Øien Sørnes, Ajay Risal, Kedar Manandhar, Hallie Thomas, Timothy J Steiner, Mattias Linde

**Affiliations:** 1 Department of Neuromedicine and Movement Science, NTNU Norwegian University of Science and Technology, Trondheim, Norway; 2 Dhulikhel Hospital, Kathmandu University Hospital, Dhulikhel, Kavre, Nepal; 3 Kathmandu University School of Medical Sciences, Dhulikhel, Kavre, Nepal; 4 Division of Brain Sciences, Imperial College London, London, UK

**Keywords:** Herbal medications, pharmacodynamic activity, overuse, South-East Asia region, Global Campaign against Headache

## Abstract

**Background:**

In Nepal, traditional treatment using medicinal plants is popular. Whereas medication-overuse headache is, by definition, caused by excessive use of acute headache medication, we hypothesized that medicinal plants, being pharmacologically active, were as likely a cause.

**Methods:**

We used data from a cross-sectional, nationwide population-based study, which enquired into headache and use of medicinal plants and allopathic medications. We searched the literature for pharmacodynamic actions of the medicinal plants.

**Results:**

Of 2100 participants, 1794 (85.4%) reported headache in the preceding year; 161 (7.7%) reported headache on ≥15 days/month, of whom 28 (17.4%) had used medicinal plants and 117 (72.7%) allopathic medication(s). Of 46 with probable medication-overuse headache, 87.0% (40/46) were using allopathic medication(s) and 13.0% (6/46) medicinal plants, a ratio of 6.7:1, higher than the overall ratio among those with headache of 4.9:1 (912/185). Of 60 plant species identified, 49 were pharmacodynamically active on the central nervous system, with various effects of likely relevance in medication-overuse headache causation.

**Conclusions:**

MPs are potentially a cause of medication-overuse headache, and not to be seen as innocent in this regard. Numbers presumptively affected in Nepal are low but not negligible. This pioneering project provides a starting point for further research to provide needed guidance on use of medicinal plants for headache.

## Background

It has been assumed that medication-overuse headache (MOH) is less of a problem in highly rural poor countries because of the lack of access to pharmaceuticals (1). However, in a large epidemiological study in Nepal, the prevalence of probable MOH (pMOH, defined as the association of headache on ≥15 days/month with overuse of acute medication) was higher than reported in Europe (2,3).

In such countries, there is often strong reliance on alternative and complementary practitioners, and the use of plants for medicinal purposes is common (4,5). If these medicinal plants (MPs) have properties that make them active against acute headache, might their overuse also generate MOH? Studies to answer this are lacking, but the question is of interest also in the Western world, where the use of MPs is increasing (6), with a widespread belief that they are “harmless remedies without side effects” (7). At the same time, MOH is the type of headache associated with the highest cost per person in Europe (8).

In a pioneering project, we used data from a large population-based survey from Nepal to test the hypothesis: “Medicinal plants, being pharmacologically active, are as likely as other medications to cause MOH”. Our public-health objectives were:

- to establish the proportion of Nepalese adults using MPs against headache
- to assess the associations of such usage with demographic factors and disease attributes (symptom burden)
- to identify the MPs used
- to make a systematic listing of their pharmacological effects on the CNS
- to ascertain whether and to what extent they might be implicated in MOH causation

## Methods

### Design of the epidemiological study

This methodology has been described in detail previously (9). In summary, in a cross-sectional, population-based survey, trained health workers visited 2210 households selected randomly from 15 representative districts out of 75 in Nepal (2,9,10). One adult was randomly selected from each household, with 2100 agreeing to participate in a structured interview. The participation proportion was > 99% (2). All data were collected during May 2013.

### Instruments

The structured *Headache-Attributed Restriction, Disability, Social Handicap and Impaired Participation* (HARDSHIP) questionnaire was culturally adapted and translated into Nepali (9). HARDSHIP included demographic factors (age, gender, household consumption and altitude and urbanicity of dwelling) as well as indices of symptom burden (headache frequency, duration and intensity) and use of allopathic medication(s). Queries concerning MPs were added (2): Participants were asked whether they had used any herbal therapies specifically to relieve headache (not taken regularly to prevent headache), their names, if so, and on how many days they had been used during the preceding month.

### Diagnosis

We specifically identified participants who reported headache on ≥15 days/month, and, among these, any who were using medications and/or MPs for acute headache. We diagnosed overuse in those who reported: a) Use on ≥15 days/month of either one type of MP or simple analgesics only; or b) use on ≥10 days/month of (i) more than one type of MP; (ii) any other acute medication (such as opioids, ergots or triptans); (iii) a combination of analgesics in different classes or of analgesics with other medications; or (iv) a combination of MPs and analgesics (2).

Migraine or tension-type headache (TTH) were diagnosed according to ICHD-3 beta criteria (11) in those with headache on < 15 days/month. Further information on the methodology has been published elsewhere (2). 

### Plant identification

Two Nepalese botanists living in Norway were consulted to correct any local plant names that were misspelled in the data and to identify the genus and species of each plant from its local name(s) and description. Synonyms were searched for at www.efloras.org (12).

### Literature search

We searched Micromedex and PubMed for reports of any pharmacological effects of the MPs on the CNS. Each plant’s botanical name was used as a search term, with the Medical Subject Heading (MeSH) term “central nervous system” added in the PubMed search. When there were fewer than three matches with the additional search term, only the plant name was used. In cases where we only had the plant-genus, “species” was added as a third search term in cases where there were >150 matches. We also looked for the plants in the encyclopaedia *Plants and people of* *Nepal* (13). When the plant names identified by the botanists gave no or few (<3) results, synonyms were searched for. We also looked for potential information sources in the reference lists of relevant articles.

We separated the potential effects of the plants on the CNS into (a) antioxidative effects, (b) anti-inflammatory or immunological effects (anti-inflammatory, pro-inflammatory, immunosuppressive, immunomodulatory effects, or influences on nitric oxide/inducible nitric oxide synthase), (c) effects on receptors, transmitters or synapses in the CNS, (d) effects on vasculature (constrictor or dilator), and (e) other effects. 

### Data management and statistical analyses

The outcome variables “use of medicinal plant” and “use of allopathic medications” during the preceding month for treatment of headache were categorised as “yes” or “no” and presented as numbers and percentages. Users of both MPs and allopathic medications were included in both categories. Proportions of participants using MPs or allopathic medications, with 95% confidence intervals (CIs), were estimated for any headache, migraine and headache on ≥15 days/month. The independent variable “age” was categorized into five groups (18–25, 26–35, 36–45, 46–55 and 56–65 years). Household consumption per year in United States dollars (USD) (at the time of study: 1 USD ≈ 100 Nepalese rupees) was used as an indicator of participants’ economic status, and categorized into three groups: Poorest (<950 USD/year), poor (950–1200 USD/year), and intermediate and above (>1200 USD/year). This gave the best split in numbers, while taking some account of Nepal’s absolute poverty line (nationally, USD 225 for an individual in 2013, but varying between locations; in Kathmandu, it was USD471 (14)). Our measure of consumption was based on households rather than individuals, and therefore set at a higher level. Dwelling was categorized as urban or rural, and altitude as low (<1000 m) or high (≥1000 m).

The symptom burden of headache was measured by frequency (headache days/month [d/m]), attack duration (hours) and headache intensity. While the first two of these were collected as continuous data, we categorized them so as to yield the best split in numbers between the categories. Thus frequency was categorized into four groups for any headache (<1, 1–2, 3–14 and ≥15 d/m), three for migraine (<1, 1–2 and 3–14 d/m) and two for headache on ≥15 d/m (15–20 and > 20 d/m). Duration was categorized into three groups: For any headache and migraine as < 4, 4–12 and > 12 hours, and for headache on ≥15 d/m as < 48, 48–144 and > 144 hours. We calculated proportion of time in ictal state (pTIS; %) from frequency and mean headache duration, and categorized it, again to give the best split in numbers, into four groups (<1, 1–3, 3.1–10 and > 10%) for any headache and migraine, and three groups (<60, 60–80 and > 80%) for headache on ≥15 days/month. Intensity was categorized as “not bad”, “quite bad” and “very bad” (equating to “mild”, “moderate” and “severe”).

We used bivariate and multivariate logistic regression analyses (with odds ratios [OR] and adjusted odds ratios [AOR], each with 95% CIs) to investigate the associations of plant or medication use on the one hand, in all headache, migraine and headache on ≥15 days/month, and demographic variables and indices of headache burden on the other. These variables were entered as covariates in the multivariate logistic regression analyses, although, for frequency and duration, pTIS was excluded, and, for pTIS, frequency and duration were excluded. 

In view of the number of analyses, we considered *p* < 0.02 to be statistically significant. Two-tailed *p*-values were calculated. All data were analysed with SPSS 21.0 software (IBM Corp, Armonk, NY, USA).

## Results

### Participants

A total of 2100 adults aged 18–65 years were included in the nationwide study; their sociodemographic characteristics have been presented previously (206). Here are analyzed data of 1794 participants (85.4%) who reported headache during the preceding year. Their mean age was 36.1 ± 12.6 years, and male/female ratio was 1:1.6.

### Use and overuse of medicinal plants and allopathic medications

Of these 1794 participants, 185 (10.3%) also reported use of MP(s) for headache at least once in the preceding month, while 912 (50.8%) had used allopathic medication(s) (Table 1). Each was more likely to be used by those with headache types expected to be more troublesome. Thus, of the 728 with migraine, 85 (11.7%) had used MP(s) and 423 (58.1%) had used allopathic medication(s) (Table 2). Of the 161 participants reporting headache on ≥15 days/month, 28 (17.4%) had used MP(s) and 117 (72.7%) had used allopathic medication(s) (Table 3). We did not separately analyse those with TTH.

**Table 1.** Use of medicinal plants and allopathic medication at least once in the preceding month among participants with any headache according to demographic factors and symptom burden (N = 1794).

Variable	N	Medicinal plants					Allopathic medication				
		n (%)	Bivariate analyses		Ethnobotany in theNepal Himalaya.Multivariate analyses		n (%)	Bivariate analyses		Multivariate analyses	
			OR (95% CI)	*p*	AOR* (95% CI)	*p*		OR (95% CI)	*p*	AOR* (95% CI)	*p*
Yes	1794	185 (10.3)	–	–	–	–	912 (50.8)	–	–	–	–
Age (in years)											
18–25	426	28 (6.6)	Reference	–	Reference	–	205 (48.1)	Reference	–	Reference	–
26–35	581	58 (10.0)	1.6 (0.9–2.5)	0.057	1.6 (0.9–2.6)	0.055	307 (52.8)	1.2 (0.9–1.6)	0.14	1.2 (0.9–1.6)	0.20
36–45	358	46 (12.8)	2.1 (1.3–3.4)	**0.003**	2.0 (1.2–3.6)	**0.006**	173 (48.3)	1.0 (0.8–1.3)	0.96	0.9 (0.7–1.3)	0.58
46–55	258	30 (11.6)	1.9 (1.1–3.2)	0.023	1.6 (0.9–2.9)	0.078	138 (53.5)	1.2 (0.9–1.7)	0.17	1.2 (0.8–1.7)	0.34
56–65	171	23 (13.5)	2.2 (1.2–3.9)	**0.008**	1.9 (1.1–3.5)	0.037	89 (52.0)	1.2 (0.8–1.7)	0.34	1.0 (0.7–1.4)	0.91
Gender											
Male	689	82 (11.9)	Reference	–	Reference	–	306 (44.4)	Reference	–	Reference	–
Female	1105	103 (9.3)	0.8 (0.6–1.1)	0.081	0.8 (0.6-1.1)	0.11	606 (54.8)	1.5 (1.31.8)	**< 0.001**	1.3 (1.1–1.6)	**0.014**
Household consumption (USD/year)											
950–1200	687	70 (10.2)	Reference	–	Reference	–	347 (50.5)	Reference	–	Reference	–
<950	686	76 (11.1)	1.1 (0.8–1.5)	0.60	0.9 (0.6–1.3)	0.67	355 (51.7)	1.1 (0.9–1.3)	0.65	1.0 (0.8–1.3)	0.97
>1200	421	39 (9.3)	0.9 (0.6–1.4)	0.62	0.9 (0.6–1.4)	0.70	210 (49.9)	1.0 (0.8–1.2)	0.85	1.1 (0.8–1.4)	0.71
Dwelling											
Urban	686	36 (5.2)	Reference	–	Reference	–	299 (43.6)	Reference	–	Reference	–
Rural	1108	149 (13.4)	2.8 (1.9–4.1)	**<0.001**	2.6 (1.8–3.9)	**<0.001**	613 (55.3)	1.6 (1.3–1.9)	**<0.001**	1.7 (1.4–2.1)	**<0.001**
Household altitude											
<1000 m	845	63 (7.5)	Reference	–	Reference	–	393 (46.5)	Reference	–	Reference	–
≥1000 m	949	122 (12.9)	1.8 (1.3–2.5)	**<0.001**	1.5 (1.1–2.2)	**0.015**	519 (54.7)	1.4 (1.2–1.7)	**0.001**	1.1 (0.9–1.4)	0.34
Headache burden indices											
Frequency (days/month)											
<1	626	46 (7.3)	Reference	–	Reference	–	203 (32.4)	Reference	–	Reference	–
1–2	531	61 (11.5)	1.6 (1.1–2.4)	**0.016**	1.5 (0.9–2.4)	0.082	285 (53.7)	2.4 (1.9–3.1)	**<0.001**	1.8 (1.3–2.3)	**<0.001**
3–14	478	50 (10.5)	1.5 (0.9–2.3)	0.067	1.2 (0.7–2.0)	0.49	307 (64.5)	3.8 (2.9–4.9)	**<0.001**	2.1 (1.6–2.9)	**<0.001**
≥15	161	28 (17.4)	2.7 (1.6–4.4)	**<0.001**	2.0 (1.1–3.8)	**0.015**	117 (72.7)	5.5 (3.8–8.1)	**<0.001**	2.3 (1.5–3.7)	**<0.001**
Duration (in hours)											
<4	590	43 (7.3)	Reference	–	Reference	–	183 (31.0)	Reference	–	Reference	–
4–12	466	47 (10.1)	1.4 (0.9–2.2)	0.11	1.2 (0.7–2.1)	0.50	227 (48.7)	2.1 (1.6–2.7)	**<0.001**	1.6 (1.2–2.1)	**0.001**
>12	738	95 (12.9)	1.9 (1.3–2.7)	**0.001**	2.2 (0.9–5.4)	0.080	502 (68.0)	4.7 (3.7–6.0)	**<0.001**	2.5 (1.8–3.4)	**<0.001**
Intensity											
Not bad	373	32 (8.6)	Reference	–	Reference	–	130 (34.9)	Reference	–	Reference	–
Quite bad	901	83 (9.2)	1.1 (0.7–1.7)	0.72	0.9 (0.6–1.4)	0.68	451 (50.1)	1.9 (1.5–2.4)	**<0.001**	1.3 (1.1–1.7)	0.047
Very bad	520	70 (13.5)	1.7 (1.1–2.6)	0.025	1.1 (0.7–1.8)	0.76	331 (63.7)	3.3 (2.5–4.3)	**<0.001**	1.7 (1.3–2.4)	**0.001**
Proportion (%) of time in ictal state											
<1	785	58 (7.4)	Reference	–	Reference	–	264 (33.6)	Reference	–	Reference	–
1–3	219	27 (12.3)	1.8 (1.1–2.9)	0.022	1.8 (1.1–3.0)	0.021	118 (53.9)	2.3 (1.7–3.1)	**<0.001**	2.2 (1.6–3.0)	**<0.001**
3.1–10	403	49 (12.2)	1.7 (1.2–2.6)	**0.007**	1.7 (1.1–2.7)	**0.013**	252 (62.5)	3.3 (2.6–4.2)	**<0.001**	3.0 (2.3–3.9)	**<0.001**
>10	387	51 (13.2)	1.9 (1.3–2.8)	**0.002**	2.0 (1.3–30)	**0.002**	278 (71.8)	5.0 (3.3–6.6)	**<0.001**	4.4 (3.3–5.9)	**<0.001**

AOR: adjusted odds ratio: OR: odds ratio; CI: confidence interval. N = total number within subsample; n = number within subsample responding positively.

* Adjusted for age, gender, household consumption, dwelling, altitude, headache frequency (F), attack duration (D), intensity and proportion of time in ictal state (pTIS) (for F and D, pTIS was excluded; for pTIS, F and D were excluded). *p*-values are two-tailed, and emboldened when significant (<0.02).

**Table 2.** Use of medicinal plants and allopathic medication at least once in the preceding month among participants with migraine according to demographic factors and symptom burden (n = 728).

Variable	N	Medicinal plants					Allopathic medication				
		n (%)	Bivariate analyses		Multivariate analyses		n (%)	Bivariate analyses		Multivariate analyses	
			OR (95% CI)	*p*	AOR* (95% CI)	*p*		OR (95% CI)	*p*	AOR* (95% CI)	*p*
Yes	728	85 (11.7)	–	–	–	–	423 (58.1)	–	–	–	–
Age (in years)											
18–25	153	9 (5.9)	Reference	–	Reference	–	88 (57.5)	Reference	–	Reference	–
26–35	241	29 (12.0)	2.2 (1.1–4.8)	0.048	2.1 (1.0–4.8)	0.063	143 (59.3)	1.1(0.7–1.6)	0.72	1.1 (0.7–1.8)	0.57
36–45	158	22 (13.9)	2.6 (1.2–5.8)	0.021	2.8 (1.2–6.6)	**0.014**	87 (55.1)	0.9 (0.6–1.4)	0.66	0.9 (0.6–1.5)	0.73
46–55	101	16 (15.8)	3.0 (1.3–7.1)	**0.012**	2.8 (1.2–6.8)	0.024	64 (63.4)	1.3 (0.8–2.1)	0.35	1.4 (0.8–2.4)	0.26
56–65	75	9 (12.0)	2.1(0.8–5.7)	0.11	2.0 (0.7–5.5)	0.18	41 (54.7)	0.9 (0.5–1.6)	0.68	0.6 (0.4–1.2)	0.15
Gender											
Male	249	35 (14.1)	Reference	–	Reference	–	141 (56.6)	Reference	–	Reference	–
Female	479	50 (10.4)	0.7 (0.5–1.1)	0.15	0.8 (0.5–1.3)	0.36	282 (58.9)	1.1 (0.8–1.5)	0.56	1.0 (0.7–1.4)	0.99
Household consumption (USD/year)											
950–1200	277	32 (11.6)	Reference	–	Reference	–	166 (59.9)	Reference	–	Reference	–
<950	289	33 (11.4)	1.0 (0.6–1.7)	0.96	0.8 (0.5–1.4)	0.38	169 (58.5)	1.3 (0.9–1.9)	0.25	0.8 (0.6–1.2)	0.37
>1200	162	20 (12.3)	1.1 (0.6–2.0)	0.80	1.2 (0.6–2.2)	0.63	88 (54.3)	1.2 (0.8–1.8)	0.39	0.8 (0.5–1.2)	0.21
Dwelling											
Urban	261	13 (5.0)	Reference	–	Reference	–	135 (51.7)	Reference	–	Reference	–
Rural	467	72 (15.4)	3.5 (1.9–6.4)	**<0.001**	3.4 (1.8–6.5)	**<0.001**	288 (61.7)	1.5 (1.1–2.0)	**0.009**	1.5 (1.1-2.1)	0.021
Household altitude											
<1000 m	287	21 (7.3)	Reference	–	Reference	–	145 (50.5)	Reference	–	Reference	–
≥1000 m	441	64 (14.5)	2.2 (1.3–3.6)	**0.004**	1.9 (1.1–3.2)	0.027	278 (63.0)	1.7 (1.2–2.3)	**0.001**	1.3 (0.9–1.9)	0.085
Headache burden indices											
Frequency (days/month)											
<1	235	23 (9.8)	Reference	–	Reference	–	92 (39.1)	Reference	–	Reference	–
1–2	226	31 (13.7)	1.5 (0.8–2.6)	0.19	1.2 (0.6–2.5)	0.62	140 (61.9)	2.5 (1.7–3.7)	**<0.001**	2.3 (1.5–3.6)	**<0.001**
3–14	267	31 (11.6)	1.2 (0.7–2.1)	0.51	0.8 (0.4–1.8)	0.58	191 (71.5)	3.9 (2.7–5.7)	**<0.001**	3.2 (1.9–5.2)	**<0.001**
Duration (in hours)											
<4	148	12 (8.1)	Reference	–	Reference	–	61 (41.2)	Reference	–	Reference	–
4–12	225	26 (11.6)	1.5 (0.7–3.0)	0.28	1.5 (0.7–3.2)	0.35	118 (52.4)	1.6 (1.1–2.4)	0.034	1.2 (0.7–1.8)	0.53
>12	355	47 (13.2)	1.7 (0.9–3.4)	0.11	1.8 (0.7–4.3)	0.20	244 (68.7)	3.1 (2.1–4.7)	**<0.001**	1.4 (0.8–2.4)	0.23
Intensity											
Not bad	60	4 (6.7)	Reference	–	Reference	–	27 (45.0)	Reference	–	Reference	–
Quite bad	354	40 (11.3)	1.8 (0.6–5.2)	0.29	1.5 (0.5–4.5)	0.47	195 (55.1)	1.5 (0.9–2.6)	0.15	1.3 (0.7–2.3)	0.40
Very bad	314	41 (13.1)	2.1 (0.7–6.1)	0.17	1.5 (0.5–4.6)	0.46	201 (64.0)	2.2 (1.2–3.8)	**0.006**	1.7 (0.9–3.1)	0.094
Proportion (%) of time in ictal state											
<1	263	24 (9.1)	Reference	–	Reference	–	111 (42.2)	Reference	–	Reference	–
1–3	83	11 (13.3)	1.5 (0.7–3.3)	0.28	1.5 (0.7–3.2)	0.34	50 (60.2)	2.1 (1.3–3.4)	**0.004**	2.2 (1.3–3.7)	**0.003**
3.1–10	248	36 (14.5)	1.7 (1.0–2.9)	0.061	1.6 (0.9–2.9)	0.12	161 (64.9)	2.5 (1.8–3.6)	**<0.001**	2.4 (1.7–3.5)	**<0.001**
>10	134	14 (10.4)	1.2 (0.6–2.3)	0.67	1.1 (0.6–2.4)	0.72	101 (75.4)	4.2 (2.6–6.6)	**<0.001**	4.4 (2.7–7.1)	**<0.001**

AOR: adjusted odds ratio; OR: odds ratio; CI: confidence interval. N = total number within subsample; n = number within subsample responding positively.

* Adjusted for age, gender, household consumption, dwelling, altitude, headache frequency (F), attack duration (D), intensity and proportion of time in ictal state (pTIS) (for F and D, pTIS was excluded; for pTIS, F and D were excluded). *p*-values are two-tailed, and emboldened when significant (<0.02).

**Table 3.** Use of medicinal plants and allopathic medication at least once in the preceding month among participants with headache on ≥ 15 days/month according to demographic factors and symptom burden (n = 161).

Variable	N	Medicinal plants					Allopathic medication				
		n (%)	Bivariate analyses		Multivariate analyses		n (%)	Bivariate analyses		Multivariate analyses	
			OR (95% CI)	*p*	AOR* (95% CI)	*p*		OR (95 % CI)	*p*	AOR* (95% CI)	*p*
Yes	161	28 (17.4)	–	–	–	–	117 (72.7)	–	–	–	–
Age (in years)											
18–25	29	3 (10.3)	Reference	–	Reference	–	17 (58.6)	Reference	–	Reference	–
26–35	51	10 (19.6)	2.1 (0.5–8.4)	0.29	2.4 (0.6–10.6)	0.23	38 (74.5)	2.1 (0.8–5.4)	0.14	1.5 (0.5–4.6)	0.47
36–45	34	9 (26.5)	3.1 (0.8–12.9)	0.12	2.6 (0.6–12.0)	0.21	24 (70.6)	1.7 (0.6–4.8)	0.32	1.4 (0.4–4.5)	0.58
46–55	29	5 (17.2)	1.8 (0.4–8.3)	0.45	2.4 (0.5–12.6)	0.29	22 (75.9)	2.2 (0.7–6.8)	0.17	1.6 (0.5–5.6)	0.47
56–65	18	1 (5.6)	0.5 (0.1–5.3)	0.57	0.6 (0.1–6.7)	0.67	16 (88.9)	5.6 (1.1–29.3)	0.039	7.8 (1.2–50.6)	0.029
Gender											
Male	44	10 (22.7)	Reference	–	Reference	–	26 (59.1)	Reference	–	Reference	–
Female	117	18 (15.4)	0.6 (0.3–1.5)	0.28	0.5 (0.2–1.6)	0.25	91 (77.8)	2.4 (1.2–5.1)	**0.019**	1.8 (0.7–4.7)	0.21
Household consumption (USD/year)											
950–1200	59	10 (16.9)	Reference	–	Reference	–	41 (69.5)	Reference	–	Reference	–
<950	59	13 (22.0)	1.4 (0.9–2.2)	0.11	1.2 (0.7–2.1)	0.50	41 (69.5)	1.0 (0.5–2.2)	1.0	1.2 (0.5–2.9)	0.75
>1200	43	5 (11.6)	1.9 (1.3–2.7)	**0.001**	2.2 (0.9–5.4)	0.080	35 (81.4)	1.9 (0.7–4.9)	0.18	1.9 (0.6–5.8)	0.25
Dwelling											
Urban	54	5 (9.3)	Reference	–	Reference	–	42 (77.8)	Reference	–	Reference	–
Rural	107	23 (21.5)	2.7 (0.9–7.5)	0.060	2.1 (0.7–6.5)	0.20	75 (70.1)	0.7 (0.3–1.4)	0.18	0.7 (0.3–1.7)	0.39
Household altitude											
<1000 m	69	12 (17.4)	Reference	–	Reference	–	50 (72.5)	Reference	–	Reference	–
≥1000 m	92	16 (17.4)	1.0 (0.4–2.3)	1.0	0.9 (0.3-2.2)	0.74	67 (72.8)	1.1 (0.5-2.1)	0.30	1.2 (0.5–2.6)	0.72
Headache burden indices											
Frequency (days/month)											
15–20	99	24 (24.2)	Reference	–	Reference	–	73 (73.7)	Reference	–	Reference	–
>20	62	4 (6.5)	0.2 (0.1–0.7)	**0.007**	0.4 (0.1–1.6)	0.18	44 (71.0)	0.9 (0.4–1.8)	0.70	0.2 (0.1–0.7)	**0.010**
Duration (in hours)											
<48	56	9 (16.1)	Reference	–	Reference	–	31 (55.4)	Reference	–	Reference	–
48–144	50	15 (30.0)	2.2 (0.9–5.7)	0.091	2.6 (0.9–7.5)	0.089	40 (80.0)	3.2 (1.4–7.7)	0.008	2.6 (1.0–6.9)	0.053
>144	55	4 (7.3)	0.4 (0.1–1.4)	0.16	0.9 (0.2–4.6)	0.91	46 (83.6)	4.1 (1.7–10.1)	**0.002**	8.6 (2.1–34.7)	**0.003**
Intensity											
Not bad/quite bad	62	9 (14.5)	Reference	–	Reference	–	41 (66.1)	Reference	–	Reference	–
Very bad	99	19 (19.2)	1.4 (0.6–3.3)	0.45	1.5 (0.6–4.0)	0.43	76 (76.8)	1.7 (0.8–3.4)	0.14	1.3 (0.6–3.1)	0.49
Proportion (%) of time in ictal state											
<60	64	14 (21.9)	Reference	–	Reference	–	46 (71.9)	Reference	–	Reference	–
60–80	45	11 (24.4)	1.2 (0.5–2.8)	0.75	1.3 (0.5–3.4)	0.61	30 (66.7)	0.8 (0.3–1.8)	0.56	0.7 (0.3–1.7)	0.68
>80	52	3 (5.8)	0.2 (0.1–0.8)	0.023	0.2 (0.1–0.9)	0.041	41 (78.8)	1.5 (0.6–3.5)	0.39	0.8 (0.3–2.2)	0.71

AOR: adjusted odds ratio: OR: odds ratio; CI: confidence interval. N = total number within subsample; n = number within subsample responding positively.

* Adjusted for age, gender, household consumption, dwelling, altitude, headache frequency (F), attack duration (D), intensity and proportion of time in ictal state (pTIS) (for F and D, pTIS was excluded; for pTIS, F and D were excluded). *p*-values are two-tailed, and emboldened when significant (<0.02).

Of the 117 participants with headache on ≥15 days/month and using allopathic medication(s), 40 (34.2%) met our criteria for overuse. Of the 28 with headache on ≥15 days/month and using MP(s), six (21.4%) met our criteria for overuse. Three of these also reported use of allopathic medication(s), one on 15 days/month and therefore meeting our criteria for overuse of these also.

### Plant identification

From the 73 local plant names reported, the botanists were able to identify or partially identify 45 plants (61.6%) and unable to identify 28. Of the 45, five were identified only by their genera, three were given only probable identities, and one was only partly identified (Herbal products Vicks). The identities of two others ‒ “banphool” and “ketaki” ‒ were not confirmed by the botanists but, in the views of the two Nepalese authors (AR and KM), very likely nonetheless, and accepted since these were relevant to the hypothesis; therefore, 47 local plant names (64.4%) in total were at least partially identified.

Four of the local plant names were applied to mixtures of different species, two were identified with more than one probable botanical name, two were used for more than one species and two for the same species as two others. Zandu balm was an exception, since this was found to contain both exact species and a mix of different species in a genus. Taken together, there were 60 species (four of them uncertain because of uncertain identifications by the botanists and six without definite confirmation by the botanists) and five genera identified from the 47 local plant names.

### Literature search

We reviewed 191 publications or encyclopaedias in the literature search. Of the 60 species, 49 were identified pharmacodynamically. Of the five genera, only two were identified pharmacodynamically since there were few studies on the genera as a whole. We found only one randomized controlled trial (RCT) relevant to use for headache, which was of low quality. No meta-analyses were found. The remainder of the studies found were animal, *in vitro*, or case studies (Table 4).

**Table 4.** Medicinal plants used for headache, with their botanical names (local names in parenthesis), pharmacodynamic properties and reported adverse events.

Formulation	Used for headache (source and level of evidence (15))	Assumed pharmacodynamic mechanisms on CNS	Reported adverse events on CNS (serious: SAEs [including death]; non-serious: NSAEs)
Abelmoschus moschatus Medik* (component of *Navaratna* oil)			
Topical (13)	No data	Antioxidative (16)	
*Abrus precatorius L.* (Rato gerdi)			
Oral/topical (13)	(Level V) (15)	Immunostimulating (17); receptors (17)	SAEs: Death (18–20); coma (18,19); seizures (18) NSAEs: Fever, reduced mental status (18)
*Aconitum ferox Wall. Ex. Ser.* (Bikma herbal)			
Oral/topical (13,18,21)	No data	Other (18,22–26); Receptors (27,28)	SAEs: Death, paralysis (18); muscular fasciculations, tonic-clonic seizures (18,25) NSAEs: Paresthesias, pain, severe headache, restlessness, apprehension, confusion, incoordination, miosis, mydriasis, diplopia, blurred vision, yellow-green vision (18)
*Acorus calamus L*. (Bojo)			
Oral/parenteral/topical (13,18)	(Level V) (18)	Other (29,30)	SAEs: None reported (18) NSAEs: None reported (18)
Allium sativum L.*** (Garlic)			
Oral/topical (13,18)	(Level V) (31)	Antioxidative (18,32–35); anti-inflammatory (36–38); receptors (33,35); other (31,39); antiapoptotic (35,36)	SAEs: None reported on CNS (18) NSAEs: Headache, fatigue, vertigo (18)
Allium wallichii Kunth*** (Garlic)			
Oral (13)	No data	Antioxidative (40)	No data
*Aloe vera L.* Burm. f. (Aloe vera herbal)			
Oral/topical (13,18,41)	No data	Antioxidative (42–45); anti-inflammatory (18,43,46); other (42,47)	SAEs: none reported (18,41) NSAEs: none reported (18,41)
*Artemisia indica* Willd. (Titepati leaves)			
Oral/topical (13)	(Level V) (48)	Antioxidative (49); immunostimulating (50); receptors (51)	No data
*Azadirachta indica* A. Juss. (Neem leaves)			
Oral/topical (13,18)	(Level V) (13)	Antioxidative (52); anti-inflammatory (53,54); receptors (55); antiapoptotic (52,53)	SAEs: Death, Reye-like syndrome, altered consciousness, seizures, decreased responsiveness (18) NSAEs: Lethargy (18)
*Brassica napus L.* (Mustard paste)			
No data	No data	No data	No data
*Calotropis gigantea L. Dryand* (Aank)			
Inhaled/oral/topical (13)	No data	Receptors (56)	No data
*Centella asiatica L.* Urb. (Gortapre)			
Oral/parenteral/topical (13,18)	(Level V) (13)	Antioxidative (57–61); anti-inflammatory (18,62); receptors (63–66); antiapoptotic (62)	No data
*Cheilanthes albomarginata* C.B. Clarke (Rani sini herbal)			
Oral/topical (13)	No data	Antioxidative (67); anti-inflammatory (67)	No data
*Colebrookea oppositifolia* Sm. (Dursur)			
Intranasal/oral/parenteral/topical (13)	(Level V) (13)	Receptors (68)	No data
*Curcuma longa L.* (Turmeric herbal)			
Oral/Parenteral (18)	No data	Antioxidative (18,69–72); anti-inflammatory (18,69–71,73–77); receptors (78–81); other (69,82–85); vascular (77)	SAEs: None reported (18,41,86) NSAEs: None reported (5,41,86)
*Curcuma zedoaria* (Christm.) Roscoe* (component of Navaratna oil)			
No data	No data	Antioxidative (87)	No data
*Cymbopogon pendulus* (Nees ex Steud.) W. Watson (Lemon grass)			
No data	No data	No data	No data
Cyperus rotundus L.* (component of Navaratna oil)			
Oral (13)	No data	Antioxidative (88,89); anti-inflammatory (89,90); receptors (91,92); other (93); antiapoptotic (89)	No data
Eclipta prostrata L.* (component of Navaratna oil)			
Oral/topical (13)	No data	Antioxidative (94); receptors (94); other (95)	No data
*Elettaria cardamomum L.* (Aalainch)			
Oral (18,41)	No data	Antioxidative (18,96); anti-inflammatory (97,98); other (99)	SAEs: None reported (18) NSAEs: None reported (18)
*Eucalyptus globulus* Labill.* (component of Zandu balm, from “Herbal products Vick”)			
Oral/parenteral/topical (18,41)	No data	Antioxidative (18); anti-inflammatory (18); receptors (100)	SAEs: Loss of consciousness, hypoventilation, convulsions (101,102) NSAEs: Ataxia, CNS depression (101)
*Gaultheria fragrantissima* Wall.* (component of Zandu balm, from “Herbal products Vick”)			
Topical (48)	(Level V) (48)	Antioxidative (103,104)	No data
*Helianthus annuus L.* (Sunflower)			
No data	No data	Antioxidative (105); anti-inflammatory (105)	No data
Hibiscus rosa-sinensis L.* (component of Navaratna oil)			
Oral/topical (13)	No data	Antioxidative (106); receptors (107)	No data
Hordeum vulgare L.* (Jamara)			
Oral/topical (18)	No data	Antioxidative (18); other (108)	SAEs: None reported (18) NSAEs: None reported (18)
*Inula cappa* (Buch.-Ham. ex D.Don) DC. (Dwareko jaro)			
Oral/topical (13)	(Level V) (2)	Anti-inflammatory (109,110)	No data
*Lilium polyphyllum* D. Don* (component of Navaratna oil)			
No data	No data	No data	No data
*Lygodium japonicum* (Thumb.) Sw.** (Pinase)			
Topical (13)	No data	Anti-inflammatory (111)	No data
*Lysimachia alternifolia* Wall.** (Pinase)			
Inhaled (13)	No data	No data	No data
Mentha arvensis L.* (component of Navaratna oil)			
Intranasal/oral/topical (18,41)	No data	Antioxidative (112,113); anti-inflammatory (113,114); receptors (113,115,116); other (114)	SAEs: None reported (41) NSAEs: None reported (41)
*Mentha spicata L.* (Menthol oil)			
Oral (13)	No data	Oxidative (110)	No data
*Micromeria biflora* (Buch.-Ham. Ex. D. Don) Benth.** (Pinase)			
Inhaled/oral/topical (13)	No data	No data	No data
*Myrica esculenta Buch*.-Ham. Ex. D. Don (Kafal ko bokra)			
Oral/topical (13,18)	(Level V) (13)	Antioxidative (117); anti-inflammatory (118)	No data
*(Neo)picrorhiza scrophulariiflora* (Pennel) D.Y. Hong (Kurki)			
Oral/topical (13)	No data	No data	No data
*Nyctanthes arbor-tristis L.* (Parijaat leaves)			
Oral/topical (13)	No data	Antioxidative (119); immunostimulating (120–122); anti-inflammatory (123); other (124)	No data
*Ocimum tenuiflorum L.* (Tulsi leaves/Kapoor*, ingredient in Banphool oil [translation not approved by botanists])			
Oral/topical (13,48)	(Level V) (48)	Antioxidative (125); anti-inflammatory (18); receptors (126–129)	No data
*Ophiocordyceps sinensis* (Berk.) G.H. Sung, J.M. Sung, Hywel-Jones & Spatafora (mushroom) (Yarsagumba)			
No data	No data	Antioxidative (130,131); anti-inflammatory (132); immunostimulating (132); other (132)	No data
Parmelia perlata* (Huds.) Ach. (component of Navaratna oil)			
No data	No data	No data	No data
Phyllanthus emblica L.* (component of Navaratna oil/Aamla, which is also a component of Banphool oil), and in monotherapy			
Oral (13)	No data	Antioxidative (133–137); anti-inflammatory (135–137); receptors (138); other (135,137,138)	No data
*Plumbago zeylanica L.* (Chitu)			
Oral/topical (13)	No data	Anti-inflammatory (139,140); receptors (141)	No data
*Rubus ellipticus Sm.* (Ainselu root)			
Oral/topical (13,18)	No data	Antioxidative (142,143)	No data
*Swertia angustifolia* Buch.-Ham. Ex D. Don** (Chiraito)			
Oral (13)	No data	No data	No data
*Swertia chirayita* Buch.-Ham. ex D. Don** (Chiraito)			
Oral/topical (13)	(Level V) (13), (Level V) (144)	Antioxidative (145); anti-inflammatory (146,147)	SAEs: None reported (144) NSAEs: No data
*Syzygium nervosum* A Cunn, ex DC. (Kyamuna ko munto)			
No data	No data	Antioxidative (148)	No data
*Terminalia chebula Retz.* (Harro)			
Oral/topical (13)	No data	Antioxidative (149,150); other (150)	No data
Triticum aestivum L.* (Jamara)			
Topical (18)	No data	Receptors (151)	SAEs: No data NSAEs: Transformation of episodic into daily headache (152)
*Vitex negundo L.* (Simali)			
Inhaled/oral/topical (13)	(Level V) (13)	Antioxidative (153,154); anti-inflammatory (154,155); receptors (155)	No data
*Zanthoxylum armatum DC.* (Timur)			
Oral/topical (13)	No data	Antioxidative (156,157); anti-inflammatory (158,159)	No data
Zea mays L.* (Jamara)			
No data	No data	Antioxidative, antiapoptotic (160)	No data
*Zingiber officinale* Roscoe (Ginger)			
Oral/topical (13,41)	(level V) (18); (level IIb) (161) (RCT)	Antioxidative (162,163); anti-inflammatory (162,164–168); receptors (169–171); other (172)	SAEs: None on CNS (161) (RCT) NSAEs: None on CNS (161) (RCT)
Genera with uncertain translations			
*Ageratum sp.* (Bherapate herbal)			
No data	No data	No data	No data
*Cotoneaster sp.* (Ghareko jaro or Ghare herbal)			
No data	No data	No data	No data
*Hedyotis sp. (*Nimaniko jadhibuti)			
No data	No data	No data	No data
Mentha sp. L.* (component of Zandu balm from “Herbal products Vick”/Satva Pudina*; component of Banphool oil [translation not approved by botanists])			
No data	No data	Anti-inflammatory (173); receptors (173)	SAEs: Coma (18) NSAEs: Ataxia, confusion, vertigo, CNS depression (18)
*Piper sp.* (Pepper)			
No data	No data	Anti-inflammatory (174)	
Species with uncertain translations			
*Asparagus racemosus* Willd.*** (Satawari [participants called it Sartawa])			
Oral (13,18)	No data	Antioxidative (175,176); receptors (176–179)	SAEs: None reported (18) NSAEs: None reported (18)
*Ipomoea carnea* Jacq. (Ajamari jhar [participants called it Ajawane])			
Topical (13)	No data	Antioxidative (180); anti-inflammatory (180); other (180)	No data.
*Justicia adhatoda L.* (Asurako or Asuro)			
Oral/topical (13)	No data	No data	No data
*Paris polyphylla* Sm.***(Satuwa [participants called it Sartawa])			
Oral/topical (13)	No data	No data	No data
Translations not approved by the botanists			
*Convolvulus pluricaulis* Choisy* (181) (component of Banphool oil (182))			
No data	No data	Antioxidative (183,184); receptors (185,186); other (184,187–190)	No data
*Nardostachys grandiflora* D.C.* (181) (component of Banphool oil (182))			
No data	No data	No data	No data
*Pandanus fascicularis* Lam. (181) (Ketaki (191))			
No data	No data	Antiinflammatory (192)	No data
*Santalum album L*.* (181) (component of Banphool oil (182))			
No data	No data	Other (193)	SAEs: CNS depression, seizures, coma (18) NSAEs: None reported (18)
*Syzygium aromaticum L.* Merrill & Perry* (component of Banphool oil (182))			
Oral/topical (18)	(Level V) (208)	Antioxidative (194,195); anti-inflammatory (18); receptors (196); other (197,198)	SAEs: Coma, seizures (199) NSAEs: Slight CNS depression (200)
*Terminalia bellirica* (Gaertn.) Roxb.* (181) (Barro (181), component of Banphool oil (182))			
No data	No data	Antioxidative (201,202); anti-inflammatory (203,204); receptors (205)	No data
Formulation	Used for headache (level of evidence (15))	Assumed pharmacodynamic mechanisms on CNS	Reported adverse events (AEs) on CNS, or death

NSAEs: non-serious adverse events; RCT: randomised controlled trial; SAEs: serious adverse events.

* Not in monotherapy.

** Two or more different species have same local name.

*** More than one possible botanical name.

Of the 60 species, 37 (61.7%) were shown in the literature to have antioxidative effects, 27 (45.0%) to have anti-inflammatory or immunosuppressive effects, and 23 (38.3%) to have effects on receptors, transmitters or synapses in the CNS (Table 4). Of the five genera, two were shown to have anti-inflammatory or immunosuppressive effects and one to have effects on receptors, transmitters or synapses in the CNS (Table 4). Fifteen species (25.0%) were mentioned in the literature as used against headache (Table 4). Serious adverse events (SAEs) affecting the CNS or resulting in death were reported for seven species (coma, loss of consciousness, seizures, paralyses, death) (Table 4). In addition, *Allium sativum* L. (garlic) has been shown to have anticoagulant properties resulting in bleeding (18). Respiratory and cardiac arrest have also been reported after ingestion of *Azadirachta indica* A. Juss (18), as has disseminated intravascular coagulopathy after ingestion of *Syzygium aromaticum* (L) Merrill & Perry (199).

Systemic bioavailability is implied by these pharmacodynamic properties. This is a key issue. We did not collect data on routes of administration to avoid overloading the already-long enquiry. While the literature indicates that many preparations of these MPs would be applied topically, others are taken orally and some are prepared for inhalation. Table 4 lists only the routes of administration potentially relevant to MOH causation.

### Associations according to headache type

#### Any headache

We found somewhat greater use of allopathic medications (AOR 1.3), but not of MPs, among females than males (Table 1). There were no clear associations with age or household consumption.

Higher proportions of participants with any headache used MPs (AOR 2.6) and allopathic medications (AOR 1.7) in rural areas than urban (Table 1). Associations with high altitude, apparent in bivariate analysis, lost significance in multivariate analysis.

Increasing headache frequency (from 1 to ≥15 days/month) was clearly associated with increasingly probable use of allopathic medication(s) (from 32.4% to 72.7%, a factor of 2.2; AOR 1.8–2.3), but, for MPs, this was so only for frequency ≥15 days/month (from 7.3% to 17.4%, a factor of 2.4; AOR 2.0; Table 1). Long-duration (AOR 2.5) and severe headache (AOR 1.7) were each associated with increased use of allopathic medication(s), but not (in multivariate analyses) of MPs (Table 1). Participants with pTIS ≥1% were more likely to have used allopathic medication(s) (AOR 2.2–4.4); a similar trend for MPs became clearly significant only when pTIS exceeded 3% (AOR 1.7–2.0; Table 1). 

#### Migraine

There were no clear associations with age, gender or household consumption.

A higher proportion of participants with migraine used MPs (AOR 3.4) in rural areas than urban, but for allopathic medications this association just lost significance in multivariate analysis (AOR 1.5; *p* = 0.021; Table 2). Associations with high altitude, apparent in bivariate analysis, once more lost significance in multivariate analysis. High frequency (AOR 3.2) and pTIS ≥1% were associated with more probable use of allopathic medications (AOR 2.2–4.4) but not of MPs (Table 2).

#### Headache on ≥15 days/month

Only one association was clear: Less use of allopathic medication(s) (AOR 0.2) in very high-frequency headache (> 20 days/month) (Table 3). A similar trend for MPs (AOR 0.2) did not survive multivariate analysis. A trend away from use of MPs (AOR 0.2) with very high pTIS (>80%) was discernible but did not meet our significance threshold (*p* > 0.02; Table 3).

#### Are medicinal plants as likely as other medications to be associated with headache on ≥15 days/month?

As noted earlier, of 912 participants with headache and using allopathic medication(s), 117 (12.8%) reported headache on ≥15 days/month with 40 of these (34.2%) meeting our criteria for overuse; of 185 using MP(s), 28 (15.1%) reported headache on ≥15 days/month, six (21.4%) meeting our criteria for overuse. The difference between these proportions was not significant (*p* = 0.259 [chi-squared]). Of the 46 with headache on ≥15 days/month and overuse, 87.0% (40/46) were using allopathic medication(s) and 13.0% (6/46) were using MP(s), a ratio of 6.7:1, somewhat higher than the overall usage ratio of 4.9:1 (912/185) (again not significant: *p* = 0.262).

## Discussion

The study found that a considerable minority (10.3%) of people with headache in Nepal had used MPs as treatment for it during the preceding month, although a much higher proportion (50.8%) had used allopathic medication(s). As might be expected, use of each was more likely (MPs: 17.4%; allopathic medication(s) 72.7%) among those with headache on ≥15 days/month. Among those with migraine in particular, use of MPs was positively associated with both rural (AOR 3.4) and high-altitude (AOR 1.9) dwelling.

Use of MPs for headache is therefore common, although less so than use of allopathic medications. Use of MPs is especially common among those with headache on ≥15 days/month, again less so than allopathic medications. Both MPs and allopathic medications were overused, according to our definitions, in association with headache on ≥15 days/month.

However, answers to the questions of whether MPs might be implicated in MOH causation, raised by our fifth objective, and, if so, whether they are as likely to cause MOH as allopathic medications, raised in our starting hypothesis, depend on evidence not only of use, overuse and association but also of a potential causal mechanism. We note, before further consideration of these, that diagnosis of MOH is presumptive, even with allopathic medications: the diagnostic criteria of ICHD-3 beta for practical reasons omit evidence of causation, relying on “Not better accounted for by another ICHD-3 diagnosis” (11). In epidemiology, this last criterion cannot be reliably applied, hence the term “probable MOH”.

With this caveat, we address these questions.

Of 60 plant species identified, 49 were pharmacodynamically active on the CNS, with effects (on CNS receptors, and anti-inflammatory and antioxidative actions) likely to be relevant in MOH causation. This is a revelation of some importance, since 8.8% of all adults aged 18–65 years in Nepal (10.3% of the 85.4% with headache) apparently use MPs for headache (although only 25.0% of these plants have been recognized in the literature as treatments used for headache, and, in a study of traditional use of MPs in Western Nepal, headache was not listed as an indication for any of the plants (207)).

The probably multiple mechanisms of MOH causation are still unclear. Every known drug used in acute headache treatment, with a range of pharmacological actions, can cause MOH when overused (208,209). The MPs used for headache by our study participants are highly pharmacologically active, many with actions similar to those of conventional medications for headache: *Inter alia*, anti-inflammatory, serotonergic and opioidergic properties, also seen in headache medications such as aspirin, NSAIDs, triptans and opioids. While we cannot (and do not) conclude that MPs are an actual cause of MOH, these findings are such that MPs, wherever they are used and overused, must be included among its potential causes.

It is worth observing that, while only six participants using MPs had headache on ≥15 days/month and fulfilled our criteria for overuse, there is no accepted definition of overuse of MPs. We placed MPs on equal terms with other acute headache medication (2), so that use of MPs of a single type on ≥15 days/month or of more than one type on ≥10 days/month was deemed to be overuse. This said, our search of the literature indicated that many of the plants used for headache had multiple, independent pharmacological actions. This made it likely that each consisted of different active substances, and more logical, therefore, to use ≥10 days/month as the overuse threshold regardless of how many types were consumed, increasing the number diagnosed as MP-overusers. However, we considered the conservative definition a better test of our hypothesis.

Use of MPs for headache escalated with headache frequency at the same rate as use of allopathic medication(s), more than doubling as frequency increased from > 1 to ≥15 days/month. As, probably, the most influential driver of medication overuse, increasing headache frequency appears to operate similarly on MPs and allopathic medications. However, people in ictal state for most of the time (pTIS > 80%) had, apparently, given up on MPs. This was not seen with allopathic medications. It is easy to speculate that people in this near-end state, with headache almost all of their time, find (or believe they find) greater benefit from allopathic medications, so that many switch allegiance. Since there was no sign of reduced use of MPs as pTIS increased up to 80%, there is no support for the alternative explanation – that risk of developing more frequent headache is higher in those who use allopathic medication(s) than in those using MPs. There are no other studies on MPs and headache to throw light on this question.

Other associations were, perhaps, of limited interest. Considerably higher proportions of participants with migraine used MPs in rural areas than urban (AOR 3.5), a finding that was not unexpected, and a reflection, probably, of urban/rural cultural difference driven, in part, by less-easy access in rural areas to health care. Association with high altitude lost significance in multivariate analysis because rural dwelling was itself strongly associated with higher altitude.

There were limitations in this study. First, even though there were 2100 participants overall, the sample size of those reporting headache on ≥15 days per month was only 161. This resulted in lower than ideal statistical power. A second limitation lay in language and plant identification. Since no data collected in the original survey related the local names of the MPs to the ethnicity of those using them, it was impossible for the botanists to identify all of them with their botanical names (more than 120 languages are spoken as mother tongue in Nepal (210)). In addition, no data had been collected on dosages or routes of administration, as already noted, or on whether the MPs were gathered by the participants themselves, prescribed by health workers or dispensed by traditional healers. Although this was information relevant to our public-health objectives, the original survey had deferred its collection to future studies (if warranted) to avoid over-loading an already lengthy enquiry.

A third factor, also limiting but outside our control, was the lack of guiding evidence in the literature. Only one relevant RCT was found in the search for side effects and use against headache, and this was of low quality. It was impossible to establish whether the MPs are actually beneficial in acute headache. Additionally, there were no studies on the pharmacological effects on the CNS for a large proportion of the MPs, which left uncertainties about their possible actions.

Despite these limitations, a participation proportion of 99.6% in the original survey excluded participation bias (2), and the methodology for collecting the data was thoroughly considered (211). These were considerable strengths. This is the first large nationwide study to investigate the use of MPs for headache in Nepal. We assume it will be the starting point for multiple endeavours: Further studies on overuse of plants with pharmacological properties among people with headache disorders; RCTs on MPs used for headache to establish their effects, if any; basic research on the mechanisms that lead to MOH to test our hypothesis more decisively, and provision of guidance on the use and misuse of MPs for headache.

## Conclusion

MPs are used as symptomatic treatments for headache by almost 9% of the Nepalese population aged 18–65 years, 15% of whom report headache on ≥15 days/month. Many of these MPs have pharmacodynamic properties similar to those implicated in MOH causation by allopathic medication overuse. On this accumulation of evidence, MPs, whenever used, should be considered at least a potential cause of MOH, perhaps no less than allopathic medications, although this is unproven. The six presumptive cases we identified in Nepal, in a sample of 2100 (0.3%), are, in public-health terms, a small but not negligible proportion. Given a global context in which herbal remedies are becoming increasingly popular, this evidence attaches considerable importance to further research on MOH in relation to MPs. This study provides a starting point, where none existed before.

## Public health relevance

- Medicinal plants must be included among the potential causes of MOH.
- In Nepal, where use of medicinal plants is culturally entrenched, presumptive evidence suggests a small but nonetheless measurable impact at population level.
- In developed countries, where MOH is the headache with the highest cost per person, the use of medicinal plants is widely believed to be harmless, and increasing.
- For these reasons, further research and guidance on use of MPs for headache are needed.

## Abbreviations

AE: adverse events; AOR: adjusted odds ratio; CI: confidence interval; CNS: central nervous system; D: duration; F: headache frequency (headache days); HARDSHIP: *Headache-Attributed Restriction, Disability, Social Handicap and Impaired Participation;* IRC-KUSMS: Institutional Review Committee of Kathmandu University School of Medical Sciences; MeSH: Medical Subject Heading; MOH: medication-overuse headache; MP: medicinal plant; NSAEs: non-serious adverse events; OR: odds ratio; pMOH: probable MOH; pTIS: proportion of time in ictal state; RCT: randomized controlled trial; SAEs: serious adverse events; TTH: tension-type headache; USD: United States dollar.

## References

[1] Jones JM. Headaches beyond the roads. Headache 2010; 50: 1219–1220. 20649656 10.1111/j.1526-4610.2010.01735.x

[2] Manandhar K Risal A Steiner TJ, et al. The prevalence of primary headache disorders in Nepal: A nationwide population-based study. J Headache Pain 2015; 16: 95. 26554602 10.1186/s10194-015-0580-y PMC4641072

[3] Stovner LJ Andree C. Prevalence of headache in Europe: A review for the Eurolight project. J Headache Pain 2010; 11: 289–299. 20473702 10.1007/s10194-010-0217-0 PMC2917556

[4] Sharma UR Malla KJ Uprety RK. Conservation and management efforts of medicinal and aromatic plants in Nepal. Banko Janakari 2017; 14; 3–11.

[5] Kunwar RM Bussmann RW. Ethnobotany in the Nepal Himalaya. J Ethnobiol Ethnomed 2008; 4: 24. 19055723 10.1186/1746-4269-4-24 PMC2639547

[6] Ekor M. The growing use of herbal medicines: issues relating to adverse reactions and challenges in monitoring safety. Front Pharmacol 2014; 4: 177. 24454289 10.3389/fphar.2013.00177 PMC3887317

[7] Furst R Zundorf I. Evidence-based phytotherapy in Europe: Where do we stand? Planta Med 2015; 81: 962–967. 25922913 10.1055/s-0035-1545948

[8] Linde M Gustavsson A Stovner LJ, et al. The cost of headache disorders in Europe: The Eurolight project. Eur J Neurol 2012; 19: 703–711. 22136117 10.1111/j.1468-1331.2011.03612.x

[9] Manandhar K Risal A Steiner TJ, et al. Estimating the prevalence and burden of major disorders of the brain in Nepal: Methodology of a nationwide population-based study. J Headache Pain 2014; 15: 52. 10.1186/1129-2377-15-52 PMC4144696 25146939

[10] Linde M Edvinsson L Manandhar K, et al. Migraine associated with altitude: Results from a population-based study in Nepal. Eur J Neurol 2017; 24: 1055–1061. 28556384 10.1111/ene.13334 PMC5518276

[11] Headache Classification Committee of the International Headache Society. The International Classification of Headache Disorders, 3rd edition (beta version). *Cephalalgia* 2013; 33: 629–808. 10.1177/0333102413485658 23771276

[12] Press JR Shrestha KK Sutton DA. Annotated checklist of the flowering plants of Nepal, http://www.efloras.org/flora_page.aspx?flora_id=110 (accessed 3 April 2018).

[13] Manandhar NP Manandhar S. Plants and people of Nepal. Portland, OR: Timber Press, 2002, pp.1–599.

[14] Pokharel T. Poverty in Nepal: Characteristics and challenges. *J Poverty Invest Dev* 2015; 11.

[15] Centre for Evidence-Based Medicine. Oxford Centre for Evidence-based Medicine – levels of evidence (March 2009), https://www.cebm.net/2009/06/oxford-centre-evidence-based-medicine-levels-evidence-march-2009/ (2009, accessed 10 June 2018).

[16] Gul MZ Bhakshu LM Ahmad F, et al. Evaluation of Abelmoschus moschatus extracts for antioxidant, free radical scavenging, antimicrobial and antiproliferative activities using in vitro assays. BMC Complement Altern Med 2011; 11: 64. 10.1186/1472-6882-11-64 PMC3201038 21849051

[17] Bhasker AS Sant B Yadav P, et al. Plant toxin abrin induced oxidative stress mediated neurodegenerative changes in mice. Neurotoxicology 2014; 44: 194–203. 25010655 10.1016/j.neuro.2014.06.015

[18] Truven Health Analytics. Micromedex® 2.0, (electronic version), http://www.micromedexsolutions.com/ (2018, accessed 22 February 2018).

[19] Sahni V Agarwal SK Singh NP, et al. Acute demyelinating encephalitis after jequirity pea ingestion (*Abrus precatorius*). Clin Toxicol (Phila) 2007; 45: 77–79. 17357388 10.1080/15563650601006116

[20] Dickers KJ Bradberry SM Rice P, et al. Abrin poisoning. Toxicol Rev 2003; 22: 137–142. 15181663 10.2165/00139709-200322030-00002

[21] Chan TY. Aconitum alkaloid poisoning related to the culinary uses of aconite roots. Toxins 2014; 6: 2605–2611. 25184557 10.3390/toxins6092605 PMC4179150

[22] Fu M Wu M Qiao Y, et al. Toxicological mechanisms of *Aconitum* alkaloids. Die Pharmazie 2006; 61: 735–741. 17020146

[23] Nyirimigabo E Xu Y Li Y, et al. A review on phytochemistry, pharmacology and toxicology studies of Aconitum. J Pharm Pharmacol 2015; 67: 1–19. 25244533 10.1111/jphp.12310

[24] Yamanaka H Doi A Ishibashi H, et al. Aconitine facilitates spontaneous transmitter release at rat ventromedial hypothalamic neurons. Brit J Pharmacol 2002; 135: 816–822. 11834630 10.1038/sj.bjp.0704517 PMC1573181

[25] Ameri A. The effects of *Aconitum* alkaloids on the central nervous system. Prog Neurobiol 1998; 56: 211–235. 9760702 10.1016/s0301-0082(98)00037-9

[26] Suk KD Yoon KC Shin JP, et al. Aconite induced myelo-optic neuropathy in a rabbit model. Korean J Ophthalmol 1994; 8: 77–82. 7853736 10.3341/kjo.1994.8.2.77

[27] Zhao XY Wang Y Li Y, et al. Songorine, a diterpenoid alkaloid of the genus *Aconitum*, is a novel GABA(A) receptor antagonist in rat brain. Neurosci Lett 2003; 337: 33–36. 12524165 10.1016/s0304-3940(02)01299-5

[28] Li TF Fan H Wang YX. *Aconitum*-derived Bulleyaconitine A exhibits antihypersensitivity through direct stimulating Dynorphin A expression in spinal microglia. J Pain 2016; 17: 530–548. 26762790 10.1016/j.jpain.2015.12.015

[29] Reddy S Rao G Shetty B, et al. Effects of *Acorus calamus* rhizome extract on the neuromodulatory system in restraint stress male rats. Turkish Neurosurg 2015; 25: 425–431. 10.5137/1019-5149.JTN.11405-14.1 26037183

[30] Hazra R Ray K Guha D. Inhibitory role of *Acorus calamus* in ferric chloride-induced epileptogenesis in rat. Hum Experim Toxicol 2007; 26: 947–953. 10.1177/0960327107087791 18375638

[31] Marschollek C Karimzadeh F Jafarian M, et al. Effects of garlic extract on spreading depression: *In vitro* and *in vivo* investigations. Nutr Neurosci 2017; 20: 127–134. 25138625 10.1179/1476830514Y.0000000148

[32] Pintana H Sripetchwandee J Supakul L, et al. Garlic extract attenuates brain mitochondrial dysfunction and cognitive deficit in obese-insulin resistant rats. *Appl Physiol Nutr Metab* 2014; 39: 1373–1379. 10.1139/apnm-2014-0255 25350296

[33] Mukherjee D Banerjee S. Learning and memory promoting effects of crude garlic extract. Indian J Experim Biol 2013; 51: 1094–1100. 24579375

[34] Brunetti L Menghini L Orlando G, et al. Antioxidant effects of garlic in young and aged rat brain *in vitro*. J Med Food 2009; 12: 1166–1169. 19857085 10.1089/jmf.2008.0176

[35] Liu SG Ren PY Wang GY, et al. Allicin protects spinal cord neurons from glutamate-induced oxidative stress through regulating the heat shock protein 70/inducible nitric oxide synthase pathway. Food Funct 2015; 6: 321–330. 25473931 10.1039/c4fo00761a

[36] Zhang B Li F Zhao W, et al. Protective effects of allicin against ischemic stroke in a rat model of middle cerebral artery occlusion. Mol Med Rep 2015; 12: 3734–3738. 26045182 10.3892/mmr.2015.3883

[37] Nillert N Pannangrong W Welbat JU, et al. Neuroprotective effects of aged garlic extract on cognitive dysfunction and neuroinflammation induced by beta-amyloid in rats. Nutrients 2017; 9: 24. 10.3390/nu9010024 PMC5295068 28054940

[38] Yun HM Ban JO Park KR, et al. *Pharmacol Ther* 2014; 142: 183–195. 10.1016/j.pharmthera.2013.12.005 24333688

[39] Thorajak P Pannangrong W Welbat JU, et al. Effects of aged garlic extract on cholinergic, glutamatergic and GABAergic systems with regard to cognitive impairment in Abeta-induced rats. Nutrients 2017; 9: 686. 10.3390/nu9070686 PMC5537801 28671572

[40] Bhandari J Muhammad B Thapa P, et al. Study of phytochemical, anti-microbial, anti-oxidant, and anti-cancer properties of *Allium wallichii*. BMC Complem Alt Med 2017; 17: 102. 10.1186/s12906-017-1622-6 PMC5299666 28178952

[41] Blumenthal MB and Werner R. *The complete German Commission E monographs*. 1st ed. Austin, TX: American Botanical Council Integrative Medicine Communications, 1998, pp.1–685.

[42] Rathor N Arora T Manocha S, et al. Anticonvulsant activity of *Aloe vera* leaf extract in acute and chronic models of epilepsy in mice. J Pharm Pharmacol 2014; 66: 477–485. 24251823 10.1111/jphp.12181

[43] Yuksel Y Guven M Kaymaz B, et al. Effects of *Aloe vera* on spinal cord ischemia-reperfusion injury of rats. J Invest Surg 2016; 29: 389–398. 27142763 10.1080/08941939.2016.1178358

[44] Lee KY Weintraub ST Yu BP. Isolation and identification of a phenolic antioxidant from *Aloe barbadensis*. Free Radic Biol Med 2000; 28: 261–265. 11281293 10.1016/s0891-5849(99)00235-x

[45] Wang Y Cao L Du G. Protective effects of *Aloe vera* extract on mitochondria of neuronal cells and rat brain. Zhongguo Zhong Yao Za Zhi 2010; 35: 364–368. 20423007 10.4268/cjcmm20100324

[46] Mirshafiey A Aghily B Namaki S, et al. Therapeutic approach by *Aloe vera* in experimental model of multiple sclerosis. Immunopharmacol Immunotoxicol 2010; 32: 410–415. 20233107 10.3109/08923970903440184

[47] Halder S Mehta AK Mediratta PK. Aloe vera improves memory and reduces depression in mice. Nutr Neurosci 2013; 16: 250–254. 23394255 10.1179/1476830512Y.0000000050

[48] The World Conservation Union (IUCN). National register of medicinal plants, https://portals.iucn.org/library/sites/library/files/documents/2000-058.pdf (2000, accessed 11 February 2020).

[49] Rashid S Rather MA Shah WA, et al. Chemical composition, antimicrobial, cytotoxic and antioxidant activities of the essential oil of *Artemisia indica* Willd. Food Chem 2013; 138: 693–700. 23265542 10.1016/j.foodchem.2012.10.102

[50] Ruwali P Ambwani TK Gautam P. *In vitro* immunomodulatory potential of *Artemisia indica* Willd. in chicken lymphocytes. Vet World 2018; 11: 80–87. 29479161 10.14202/vetworld.2018.80-87 PMC5813516

[51] Khan I Karim N Ahmad W, et al. GABA-A receptor modulation and anticonvulsant, anxiolytic, and antidepressant activities of constituents from *Artemisia indica* Linn. Evid-based Complem Alt Med 2016; 2016: 1215393. 10.1155/2016/1215393 PMC4838807 27143980

[52] Vaibhav K Shrivastava P Khan A, et al. Azadirachta indica mitigates behavioral impairments, oxidative damage, histological alterations and apoptosis in focal cerebral ischemia-reperfusion model of rats. Neurol Sci 2013; 34: 1321–1330. 23187787 10.1007/s10072-012-1238-z

[53] Bedri S Khalil EA Khalid SA, et al. *Azadirachta indica* ethanolic extract protects neurons from apoptosis and mitigates brain swelling in experimental cerebral malaria. Malar J 2013; 12: 298. 23984986 10.1186/1475-2875-12-298 PMC3844317

[54] Yanpallewar S Rai S Kumar M, et al. Neuroprotective effect of *Azadirachta indica* on cerebral post-ischemic reperfusion and hypoperfusion in rats. Life Sci 2005; 76: 1325–1338. 15670613 10.1016/j.lfs.2004.06.029

[55] Pillai NR Santhakumari G Laping J. Some pharmacological actions of ‘nimbidin’ – a bitter principle of *Azadirachta indica*-a juss (neem). Anc Sci Life 1984; 4: 88–95. 22557456 PMC3331505

[56] Argal A Pathak AK. CNS activity of *Calotropis gigantea* roots. J Ethnopharmacol 2006; 106: 142–145. 16446065 10.1016/j.jep.2005.12.024

[57] Doknark S Mingmalairak S Vattanajun A, et al. Study of ameliorating effects of ethanolic extract of *Centella asiatica* on learning and memory deficit in animal models. J Med Assoc Thailand 2014; 97: S68–S76. 25518178

[58] Gray NE Sampath H Zweig JA, et al. *Centella asiatica* attenuates amyloid-beta-induced oxidative stress and mitochondrial dysfunction. J Alzheimer’s Dis 2015; 45: 933–946. 25633675 10.3233/JAD-142217 PMC4412033

[59] Dhanasekaran M Holcomb LA Hitt AR, et al. *Centella asiatica* extract selectively decreases amyloid beta levels in hippocampus of Alzheimer’s disease animal model. Phytother Res 2009; 23: 14–19. 19048607 10.1002/ptr.2405

[60] Ramanathan M Sivakumar S Anandvijayakumar PR, et al. Neuroprotective evaluation of standardized extract of *Centella asciatica* in monosodium glutamate treated rats. Indian J Experim Biol 2007; 45: 425–431. 17569283

[61] Gray NE Harris CJ Quinn JF, et al. *Centella asiatica* modulates antioxidant and mitochondrial pathways and improves cognitive function in mice. J Ethnopharmacol 2016; 180: 78–86. 26785167 10.1016/j.jep.2016.01.013 PMC4764102

[62] Song D Jiang X Liu Y, et al. Asiaticoside attenuates cell growth inhibition and apoptosis induced by Abeta1-42 via inhibiting the TLR4/NF-kappaB signaling pathway in human brain microvascular endothelial cells. Front Pharmacol 2018; 9: 28. 29441018 10.3389/fphar.2018.00028 PMC5797575

[63] Chatterjee TK Chakraborty A Pathak M, et al. Effects of plant extract *Centella asiatica* (Linn.) on cold restraint stress ulcer in rats. Indian J Experim Biol 1992; 30: 889–891. 1293014

[64] Nasir MN Abdullah J Habsah M, et al. Inhibitory effect of Asiatic acid on acetylcholinesterase, excitatory post synaptic potential and locomotor activity. Phytomedicine 2012; 19: 311–316. 22112723 10.1016/j.phymed.2011.10.004

[65] Visweswari G Prasad KS Chetan PS, et al. Evaluation of the anticonvulsant effect of *Centella asiatica* (gotu kola) in pentylenetetrazol-induced seizures with respect to cholinergic neurotransmission. Epilepsy Behav 2010; 17: 332–335. 20144879 10.1016/j.yebeh.2010.01.002

[66] Awad R Levac D Cybulska P, et al. Effects of traditionally used anxiolytic botanicals on enzymes of the gamma-aminobutyric acid (GABA) system. Can J Physiol Pharmacol 2007; 85: 933–942. 18066140 10.1139/Y07-083

[67] Lamichhane R Kim SG Poudel A, et al. Evaluation of *in vitro* and *in vivo* biological activities of *Cheilanthes albomarginata* Clarke. BMC Complement Altern Med 2014; 14: 342. 25238673 10.1186/1472-6882-14-342 PMC4180312

[68] Viswanatha GL Venkataranganna MV Prasad NBL. Ameliorative potential of *Colebrookea oppositifolia* methanolic root extract against experimental models of epilepsy: Possible role of GABA mediated mechanism. Biomed Pharmacother 2017; 90: 455–465. 28391167 10.1016/j.biopha.2017.03.078

[69] Mythri RB Bharath MM. Curcumin: A potential neuroprotective agent in Parkinson’s disease. Curr Pharmaceut Des 2012; 18: 91–99. 10.2174/138161212798918995 22211691

[70] Yu S Wang X He X, et al. Curcumin exerts anti-inflammatory and antioxidative properties in 1-methyl-4-phenylpyridinium ion (MPP(+))-stimulated mesencephalic astrocytes by interference with TLR4 and downstream signaling pathway. Cell Stress Chap 2016; 21: 697–705. 10.1007/s12192-016-0695-3 PMC4908001 27164829

[71] Wang XS Zhang ZR Zhang MM, et al. Neuroprotective properties of curcumin in toxin-base animal models of Parkinson’s disease: A systematic experiment literatures review. BMC Complement Altern Med 2017; 17: 412. 28818104 10.1186/s12906-017-1922-x PMC5561616

[72] Ma XW Guo RY. Dose-dependent effect of *Curcuma longa* for the treatment of Parkinson’s disease. Experim Ther Med 2017; 13: 1799–1805. 10.3892/etm.2017.4225 PMC5443238 28565770

[73] Ammon HPT Wahl MA. Pharmacology of *Curcuma longa*. Planta Medica 1991; 57: 1–7. 2062949 10.1055/s-2006-960004

[74] Witkin JM Li X. Curcumin, an active constituent of the ancient medicinal herb *Curcuma longa* L.: Some uses and the establishment and biological basis of medical efficacy. CNS Neurol Dis Drug Targets 2013; 12: 487–497. 10.2174/1871527311312040007 23574161

[75] Xie L Li XK Funeshima-Fuji N, et al. Amelioration of experimental autoimmune encephalomyelitis by curcumin treatment through inhibition of IL-17 production. Int Immunopharmacol 2009; 9: 575–581. 19539560 10.1016/j.intimp.2009.01.025

[76] Yu SY Gao R Zhang L, et al. Curcumin ameliorates ethanol-induced memory deficits and enhanced brain nitric oxide synthase activity in mice. Prog Neuro-Psychopharmacol Biol Psych 2013; 44: 210–216. 10.1016/j.pnpbp.2013.03.001 23500667

[77] Jiang J Wang W Sun YJ, et al. Neuroprotective effect of curcumin on focal cerebral ischemic rats by preventing blood-brain barrier damage. Eur J Pharmacol 2007; 561: 54–62. 17303117 10.1016/j.ejphar.2006.12.028

[78] Pyrzanowska J Piechal A Blecharz-Klin K, et al. The influence of the long-term administration of *Curcuma longa* extract on learning and spatial memory as well as the concentration of brain neurotransmitters and level of plasma corticosterone in aged rats. Pharmacol Biochem Behav 2010; 95: 351–358. 20219527 10.1016/j.pbb.2010.02.013

[79] Yu ZF Kong LD Chen Y. Antidepressant activity of aqueous extracts of *Curcuma longa* in mice. J Ethnopharmacol 2002; 83: 161–165. 12413724 10.1016/s0378-8741(02)00211-8

[80] Zhao X Wang C Zhang JF, et al. Chronic curcumin treatment normalizes depression-like behaviors in mice with mononeuropathy: Involvement of supraspinal serotonergic system and GABAA receptor. Psychopharmacology 2014; 231: 2171–2187. 24297305 10.1007/s00213-013-3368-2

[81] Lin TY Lu CW Wang CC, et al. Curcumin inhibits glutamate release in nerve terminals from rat prefrontal cortex: possible relevance to its antidepressant mechanism. Prog Neuro-Psychopharmacol Biol Psych 2011; 35: 1785–1793. 10.1016/j.pnpbp.2011.06.012 21741425

[82] Nabiuni M Nazari Z Safaeinejad Z, et al. Curcumin downregulates aquaporin-1 expression in cultured rat choroid plexus cells. J Med Food 2013; 16: 504–510. 23735000 10.1089/jmf.2012.0208

[83] Kim DS Kim JY Han Y. Curcuminoids in neurodegenerative diseases. Rec Pat CNS Drug Discov 2012; 7: 184–204. 10.2174/157488912803252032 22742420

[84] Nam SM Choi JH Yoo DY, et al. Effects of curcumin (*Curcuma longa*) on learning and spatial memory as well as cell proliferation and neuroblast differentiation in adult and aged mice by upregulating brain-derived neurotrophic factor and CREB signaling. J Med Food 2014; 17: 641–649. 24712702 10.1089/jmf.2013.2965 PMC4060834

[85] Li Y Li J Li S, et al. Curcumin attenuates glutamate neurotoxicity in the hippocampus by suppression of ER stress-associated TXNIP/NLRP3 inflammasome activation in a manner dependent on AMPK. Toxicol Applied Pharmacol 2015; 286: 53–63. 10.1016/j.taap.2015.03.010 25791922

[86] Goel A Kunnumakkara AB Aggarwal BB. Curcumin as “curecumin”: From kitchen to clinic. Biochem Pharmacol 2008; 75: 787–809. 17900536 10.1016/j.bcp.2007.08.016

[87] Toda S. Inhibitory effects of aromatic herbs on lipid peroxidation and protein oxidative modification of mice brain homogenate by copper in vitro. Phytother Res 2001; 15: 541–543. 11536388 10.1002/ptr.848

[88] Sunil AG Kesavanarayanan KS Kalaivani P, et al. Total oligomeric flavonoids of *Cyperus rotundus* ameliorates neurological deficits, excitotoxicity and behavioral alterations induced by cerebral ischemic-reperfusion injury in rats. Brain Res Bull 2011; 84: 394–405. 21272618 10.1016/j.brainresbull.2011.01.008

[89] Lee CH Hwang DS Kim HG, et al. Protective effect of *Cyperi rhizoma* against 6-hydroxydopamine-induced neuronal damage. J Med Food 2010; 13: 564–571. 20521982 10.1089/jmf.2009.1252

[90] Azimi A Ghaffari SM Riazi GH, et al. Alpha-cyperone of *Cyperus rotundus* is an effective candidate for reduction of inflammation by destabilization of microtubule fibers in brain. J Ethnopharmacol 2016; 194: 219–227. 27353867 10.1016/j.jep.2016.06.058

[91] Ha JH Lee KY Choi HC, et al. Modulation of radioligand binding to the GABA(A)-benzodiazepine receptor complex by a new component from *Cyperus rotundus*. Biol Pharm Bull 2002; 25: 128–130. 11824542 10.1248/bpb.25.128

[92] Pal D Dutta S Sarkar A. Evaluation of CNS activities of ethanol extract of roots and rhizomes of *Cyperus rotundus* in mice. Acta Pol Pharm 2009; 66: 535–541. 19894649

[93] Ngamrojanavanich N Manakit S Pornpakakul S, et al. Inhibitory effects of selected Thai medicinal plants on Na^+^,K^+^-ATPase. Fitoterapia 2006; 77: 481–483. 16860494 10.1016/j.fitote.2006.06.003

[94] Kim DI Lee SH Hong JH, et al. The butanol fraction of *Eclipta prostrata* (Linn) increases the formation of brain acetylcholine and decreases oxidative stress in the brain and serum of cesarean-derived rats. Nutr Res 2010; 30: 579–584. 20851313 10.1016/j.nutres.2010.08.001

[95] Jung WY Kim H Park HJ, et al. The ethanolic extract of the *Eclipta prostrata* L. ameliorates the cognitive impairment in mice induced by scopolamine. J Ethnopharmacol 2016; 190: 165–173. 27267831 10.1016/j.jep.2016.06.010

[96] Verma SK Jain V Katewa SS. Blood pressure lowering, fibrinolysis enhancing and antioxidant activities of cardamom (*Elettaria cardamomum*). Indian J Biochem Biophys 2009; 46: 503–506. 20361714

[97] Majdalawieh AF Carr RI. In vitro investigation of the potential immunomodulatory and anti-cancer activities of black pepper (*Piper nigrum*) and cardamom (*Elettaria cardamomum*). J Med Food 2010; 13: 371–381. 20210607 10.1089/jmf.2009.1131

[98] Daoudi A Aarab L Abdel-Sattar E. Screening of immunomodulatory activity of total and protein extracts of some Moroccan medicinal plants. Toxicol Ind Health 2013; 29: 245–253. 22301818 10.1177/0748233711430972

[99] Masoumi-Ardakani Y Mahmoudvand H Mirzaei A, et al. The effect of *Elettaria cardamomum* extract on anxiety-like behavior in a rat model of post-traumatic stress disorder. Biomed Pharmacother 2017; 87: 489–495. 28073098 10.1016/j.biopha.2016.12.116

[100] Girish C Raj V Arya J, et al. Evidence for the involvement of the monoaminergic system, but not the opioid system in the antidepressant-like activity of ellagic acid in mice. Eur J Pharmacol 2012; 682: 118–125. 22387858 10.1016/j.ejphar.2012.02.034

[101] Tibballs J. Clinical effects and management of eucalyptus oil ingestion in infants and young children. Med J Australia 1995; 163: 177–180. 7651249 10.5694/j.1326-5377.1995.tb124516.x

[102] Patel S Wiggins J. Eucalyptus oil poisoning. Arch Dis Child 1980; 55: 405–406. 7436478 10.1136/adc.55.5.405 PMC1626858

[103] Pandey BP Thapa R Upreti A. Chemical composition, antioxidant and antibacterial activities of essential oil and methanol extract of *Artemisia vulgaris* and *Gaultheria fragrantissima* collected from Nepal. Asian Pacific J Trop Med 2017; 10: 952–959. 10.1016/j.apjtm.2017.09.005 29111190

[104] Cong F Joshi KR Devkota HP, et al. Dhasingreoside: New flavonoid from the stems and leaves of *Gaultheria fragrantissima*. Nat Prod Res 2015; 29: 1442–1448. 25622517 10.1080/14786419.2014.1003932

[105] Guo S Ge Y Na Jom K. A review of phytochemistry, metabolite changes, and medicinal uses of the common sunflower seed and sprouts (*Helianthus annuus* L.). Chem Cent J 2017; 11: 95. 29086881 10.1186/s13065-017-0328-7 PMC5622016

[106] Nade VS Dwivedi S Kawale LA, et al. Effect of *Hibiscus rosa sinensis* on reserpine-induced neurobehavioral and biochemical alterations in rats. Indian J Exp Biol 2009; 47: 559–563. 19761039

[107] Begum Z Younus I. Hibiscus rosa sinensis mediate anxiolytic effect via modulation of ionotropic GABA-A receptors: possible mechanism of action. Metab Brain Dis 2018; 33: 823–827. 10.1007/s11011-018-0188-4 29372452

[108] Pichiah PB Cho SH Han SK, et al. Fermented barley supplementation modulates the expression of hypothalamic genes and reduces energy intake and weight gain in rats. J Med Food 2016; 19: 418–426. 27074621 10.1089/jmf.2015.3600

[109] Wu J Tang C Yao S, et al. Anti-inflammatory inositol derivatives from the whole plant of *Inula cappa*. J Nat Prod 2015; 78: 2332–2338. 26444098 10.1021/acs.jnatprod.5b00135

[110] Kalola J Shah R Patel A, et al. Anti-inflammatory and immunomodulatory activities of *Inula cappa* roots (Compositae). J Complement Integr Med 2017. DOI: /j/jcim.2017.14.issue-3/jcim-2016-0083/jcim-2016-0083.xml. 10.1515/jcim-2016-0083 28889730

[111] Cho YC Kim BR Le HTT, et al. Antiinflammatory effects on murine macrophages of ethanol extracts of *Lygodium japonicum* spores via inhibition of NFkappaB and p38. Mol Med Rep 2017; 16: 4362–4370. 29067444 10.3892/mmr.2017.7070

[112] Lin KH Yang YY Yang CM, et al. Antioxidant activity of herbaceous plant extracts protect against hydrogen peroxide-induced DNA damage in human lymphocytes. BMC Res Notes 2013; 6: 490. 24279749 10.1186/1756-0500-6-490 PMC4222091

[113] Tian W Akanda MR Islam A, et al. The anti-stress effect of *Mentha arvensis* in immobilized rats. Int J Mol Sci 2018; 19: 355. 10.3390/ijms19020355 PMC5855577 29370076

[114] Verma SM Arora H Dubey R. Anti-inflammatory and sedative – hypnotic activity of the methanolic extract of the leaves of *Mentha arvensis*. Anc Sci Life 2003; 23: 95–99. 22557118 PMC3330963

[115] Oinonen PP Jokela JK Hatakka AI, et al. Linarin, a selective acetylcholinesterase inhibitor from *Mentha arvensis*. Fitoterapia 2006; 77: 429–434. 16815640 10.1016/j.fitote.2006.05.002

[116] Feng X Wang X Liu Y, et al. Linarin inhibits the acetylcholinesterase activity *in-vitro* and *ex-vivo*. Iran J Pharm Res 2015; 14: 949–954. 26330885 PMC4518125

[117] Rawat S Jugran A Giri L, et al. Assessment of antioxidant properties in fruits of *Myrica esculenta*: A popular wild edible species in Indian Himalayan region. Evid-Based Complem Alt Med 2011; 2011: 512787. 10.1093/ecam/neq055 PMC3135792 21785629

[118] Agnihotri S Wakode S Ali M. Essential oil of *Myrica esculenta* Buch. Ham.: Composition, antimicrobial and topical anti-inflammatory activities. Nat Prod Res 2012; 26: 2266–2269. 22260222 10.1080/14786419.2011.652959

[119] Michael JS Kalirajan A Padmalatha C, et al. *In vitro* antioxidant evaluation and total phenolics of methanolic leaf extracts of *Nyctanthes arbor-tristis* L. Chinese J Nat Med 2013; 11: 484–487. 10.1016/S1875-5364(13)60088-6 24359771

[120] Agrawal J Pal A. *Nyctanthes arbor-tristis* Linn – a critical ethnopharmacological review. J Ethnopharmacol 2013; 146: 645–658. 23376280 10.1016/j.jep.2013.01.024

[121] Puri A Saxena R Saxena RP, et al. Immunostimulant activity of *Nyctanthes arbor-tristis* L. J Ethnopharmacol 1994; 42: 31–37. 8046941 10.1016/0378-8741(94)90020-5

[122] Bharshiv CK Garg SK Bhatia AK. Immunomodulatory activity of aqueous extract of *Nyctanthes arbor-tristis* flowers with particular reference to splenocytes proliferation and cytokines induction. Indian J Pharmacol 2016; 48: 412–417. 27756953 10.4103/0253-7613.186210 PMC4980930

[123] Kakoti BB Pradhan P Borah S, et al. Analgesic and anti-inflammatory activities of the methanolic stem bark extract of *Nyctanthes arbor-tristis* linn. BioMed Res Int 2013; 2013: 826295. 23984409 10.1155/2013/826295 PMC3745914

[124] Saxena RS Gupta B Lata S. Tranquilizing, antihistaminic and purgative activity of *Nyctanthes arbor tristis* leaf extract. J Ethnopharmacol 2002; 81: 321–325. 12127232 10.1016/s0378-8741(02)00088-0

[125] Balanehru S Nagarajan B. Intervention of Adriamycin induced free radical damage. *Biochem Int* 1992; 28: 735–744. 1482409

[126] Archana R Namasivayam A. Effect of *Ocimum sanctum* on noise induced changes in neutrophil functions. J Ethnopharmacol 2000; 73: 81–85. 11025142 10.1016/s0378-8741(00)00281-6

[127] Sakina MR Dandiya PC Hamdard ME, et al. Preliminary psychopharmacological evaluation of *Ocimum sanctum* leaf extract. J Ethnopharmacol 1990; 28: 143–150. 2329804 10.1016/0378-8741(90)90023-m

[128] Sembulingam K Sembulingam P Namasivayam A. Effect of *Ocimum sanctum* Linn on the changes in central cholinergic system induced by acute noise stress. J Ethnopharmacol 2005; 96: 477–482. 15619567 10.1016/j.jep.2004.09.047

[129] Jothie Richard E Illuri R Bethapudi B, et al. Anti-stress activity of *Ocimum sanctum*: Possible effects on hypothalamic-pituitary-adrenal axis. Phytother Res 2016; 30: 805–814. 26899341 10.1002/ptr.5584

[130] Pal M Bhardwaj A Manickam M, et al. Protective efficacy of the caterpillar mushroom, *Ophiocordyceps sinensis* (Ascomycetes), from India in neuronal hippocampal cells against hypoxia. Int J Med Mushrooms 2015; 17: 829–840. 26756295 10.1615/intjmedmushrooms.v17.i9.30

[131] Zhu JS Halpern GM Jones K. The scientific rediscovery of an ancient Chinese herbal medicine: *Cordyceps sinensis* part I. J Altern Complement Med 1998; 4: 289–303. 9764768 10.1089/acm.1998.4.3-289

[132] Zhu JS Halpern GM Jones K. The scientific rediscovery of a precious ancient Chinese herbal regimen: *Cordyceps sinensis*: Part II. J Altern Complement Med 1998; 4: 429–457. 9884180 10.1089/acm.1998.4.429

[133] D’Souza JJ D’Souza P P Fazal F, et al. Anti-diabetic effects of the Indian indigenous fruit *Emblica officinalis* Gaertn: Active constituents and modes of action. Food Funct 2014; 5: 635–644. 24577384 10.1039/c3fo60366k

[134] Yang B Liu P. Composition and biological activities of hydrolyzable tannins of fruits of *Phyllanthus emblica*. J Agric Food Chem 2014; 62: 529–541. 24369850 10.1021/jf404703k

[135] Variya BC Bakrania AK Patel SS. *Emblica officinalis* (Amla): A review for its phytochemistry, ethnomedicinal uses and medicinal potentials with respect to molecular mechanisms. Pharmacol Res 2016; 111: 180–200. 27320046 10.1016/j.phrs.2016.06.013

[136] Golechha M Sarangal V Ojha S, et al. Anti-inflammatory effect of *Emblica officinalis* in rodent models of acute and chronic inflammation: Involvement of possible mechanisms. Int J Inflam 2014; 2014: 178408. 25215258 10.1155/2014/178408 PMC4158298

[137] Golechha M Bhatia J Ojha S, et al. Hydroalcoholic extract of *Emblica officinalis* protects against kainic acid-induced status epilepticus in rats: Evidence for an antioxidant, anti-inflammatory, and neuroprotective intervention. Pharm Biol 2011; 49: 1128–1136. 21749189 10.3109/13880209.2011.571264

[138] Vasudevan M Parle M. Memory enhancing activity of Anwala churna (*Emblica officinalis* Gaertn.): An Ayurvedic preparation. Physiol Behav 2007; 91: 46–54. 17343883 10.1016/j.physbeh.2007.01.016

[139] Zhang K Ge Z Da Y, et al. Plumbagin suppresses dendritic cell functions and alleviates experimental autoimmune encephalomyelitis. J Neuroimmunol 2014; 273: 42–52. 24953531 10.1016/j.jneuroim.2014.05.014

[140] Jia Y Jing J Bai Y, et al. Amelioration of experimental autoimmune encephalomyelitis by plumbagin through down-regulation of JAK-STAT and NF-kappaB signaling pathways. PloS ONE 2011; 6: e27006. 22066025 10.1371/journal.pone.0027006 PMC3205001

[141] Bopaiah CP Pradhan N. Central nervous system stimulatory action from the root extract of *Plumbago zeylanica* in rats. Phytother Res 2001; 15: 153–156. 11268117 10.1002/ptr.702

[142] George BP Parimelazhagan T Kumar YT, et al. Antitumor and wound healing properties of *Rubus ellipticus* Smith. J Acupunct Merid Studies 2015; 8: 134–141. 10.1016/j.jams.2013.10.002 26100067

[143] Sharma US Kumar A. *In vitro* antioxidant activity of *Rubus ellipticus* fruits. J Adv Pharm Technol Res 2011; 2: 47–50. 22171292 10.4103/2231-4040.79805 PMC3217685

[144] Kumar V Van Staden J. A review of *Swertia chirayita* (Gentianaceae) as a traditional medicinal plant. Front Pharmacol 2015; 6: 308. 10.3389/fphar.2015.00308 PMC4709473 26793105

[145] Li J Zhao YL Huang HY, et al. Phytochemistry and pharmacological activities of the genus *Swertia* (Gentianaceae): A review. Am J Chinese Med 2017; 45: 667–736. 10.1142/S0192415X17500380 28490237

[146] Banerjee S Sur TK Mandal S, et al. Assessment of the anti-inflammatory effects of *Swertia chirata* in acute and chronic experimental models in male albino rats Indian J Pharmacol 2000; 32: 21–24.

[147] Das CS Bhadra S Roy S, et al. Analgesic and anti-inflammatory activities of ethanolic root extract of *Swertia chirata* (Gentianaceae). Jordan J Biol Sci 2012; 5: 31–36.

[148] Sukprasansap M Chanvorachote P Tencomnao T. *Cleistocalyx nervosum* var. paniala berry fruit protects neurotoxicity against endoplasmic reticulum stress-induced apoptosis. Food Chem Toxicol 2017; 103: 279–288. 28315776 10.1016/j.fct.2017.03.025

[149] Khan A Nazar H Sabir SM, et al. Antioxidant activity and inhibitory effect of some commonly used medicinal plants against lipid per-oxidation in mice brain. *African J Trad Complem Alt Med* 2014; 11: 83–90. 10.4314/ajtcam.v11i5.14 PMC4202523 25395710

[150] Park JH Joo HS Yoo KY, et al. Extract from *Terminalia chebula* seeds protect against experimental ischemic neuronal damage via maintaining SODs and BDNF levels. Neurochem Res 2011; 36: 2043–2050. 21667226 10.1007/s11064-011-0528-9

[151] Huebner FR Lieberman KW Rubino RP, et al. Demonstration of high opioid-like activity in isolated peptides from wheat gluten hydrolysates. Peptides 1984; 5: 1139–1147. 6099562 10.1016/0196-9781(84)90180-3

[152] Pascual J Leno C. A woman with daily headaches. J Headache Pain 2005; 6: 91–92. 16362649 10.1007/s10194-005-0158-1 PMC3452314

[153] Umamaheswari M Asokkumar K Umamageswari N, et al. Protective effect of the leaves of *Vitex negundo* against ethanol-induced cerebral oxidative stress in rats. Tanzania J Health Res 2012; 14: 21–28. 10.4314/thrb.v14i1.5 26591743

[154] Zheng CJ Li HQ Ren SC, et al. Phytochemical and pharmacological profile of *Vitex negundo*. Phytother Res 2015; 29: 633–647. 25641408 10.1002/ptr.5303

[155] Chan EWC Wong SK Chan HT. Casticin from *Vitex* species: A short review on its anticancer and anti-inflammatory properties. J Integrat Med 2018; 16: 147–152; 10.1016/j.joim.2018.03.001 29559215

[156] Wazir A Mehjabeen Jahan N, et al. Antibacterial, antifungal and antioxidant activities of some medicinal plants. Pak J Pharm Sci 2014; 27: 2145–2152. 26045377

[157] Ahmed S Shakeel F . Voltammetric determination of antioxidant character in *Berberis lycium* Royel, *Zanthoxylum armatum* and *Morus nigra* Linn plants. Pak J Pharm Sci 2012; 25: 501–507. 22713934

[158] Nooreen Z Kumar A Bawankule DU, et al. New chemical constituents from the fruits of *Zanthoxylum armatum* and its *in vitro* anti-inflammatory profile. Nat Prod Res 2017: 1–8. 10.1080/14786419.2017.1405404 29161887

[159] Guo T Deng YX Xie H, et al. Antinociceptive and anti-inflammatory activities of ethyl acetate fraction from *Zanthoxylum armatum* in mice. Fitoterapia 2011; 82: 347–351. 21059381 10.1016/j.fitote.2010.11.004

[160] Choi DJ Kim SL Choi JW, et al. Neuroprotective effects of corn silk maysin via inhibition of H_2_O_2_-induced apoptotic cell death in SK-N-MC cells. Life Sci 2014; 109: 57–64. 24928367 10.1016/j.lfs.2014.05.020

[161] Maghbooli M Golipour F Moghimi Esfandabadi A, et al. Comparison between the efficacy of ginger and sumatriptan in the ablative treatment of the common migraine. Phytother Res 2014; 28: 412–415. 23657930 10.1002/ptr.4996

[162] Semwal RB Semwal DK Combrinck S, et al. Gingerols and shogaols: Important nutraceutical principles from ginger. Phytochemistry 2015; 117: 554–568. 26228533 10.1016/j.phytochem.2015.07.012

[163] Otunola GA Oloyede OB Oladiji AT, et al. Selected spices and their combination modulate hypercholesterolemia-induced oxidative stress in experimental rats. Biol Res 2014; 47: 5. 25027235 10.1186/0717-6287-47-5 PMC4060372

[164] Ho SC Chang KS Lin CC. Anti-neuroinflammatory capacity of fresh ginger is attributed mainly to 10-gingerol. Food Chem 2013; 141: 3183–3191. 23871076 10.1016/j.foodchem.2013.06.010

[165] Deng XY Xue JS Li HY, et al. Geraniol produces antidepressant-like effects in a chronic unpredictable mild stress mice model. Physiol Behav 2015; 152: 264–271. 26454213 10.1016/j.physbeh.2015.10.008

[166] Grzanna R Phan P Polotsky A, et al. Ginger extract inhibits beta-amyloid peptide-induced cytokine and chemokine expression in cultured THP-1 monocytes. J Alt Complem Med 2004; 10: 1009–1013. 10.1089/acm.2004.10.1009 15673995

[167] Jafarzadeh A Arabi Z Ahangar-Parvin R, et al. Ginger extract modulates the expression of chemokines CCL20 and CCL22 and their receptors (CCR6 and CCR4) in the central nervous system of mice with experimental autoimmune encephalomyelitis. Drug Res 2017; 67: 632–639. 10.1055/s-0043-113455 28672408

[168] Park G Kim HG Ju MS, et al. 6-Shogaol, an active compound of ginger, protects dopaminergic neurons in Parkinson’s disease models via anti-neuroinflammation. Acta Pharmacol Sinica 2013; 34: 1131–1139. 10.1038/aps.2013.57 PMC4003157 23811724

[169] Lim S Moon M Oh H, et al. Ginger improves cognitive function via NGF-induced ERK/CREB activation in the hippocampus of the mouse. J Nutr Biochem 2014; 25: 1058–1065. 25049196 10.1016/j.jnutbio.2014.05.009

[170] Qian QH Yue W Chen WH, et al. Effect of gingerol on substance P and NK1 receptor expression in a vomiting model of mink. Chinese Med J 2010; 123: 478–484. 20193490

[171] Gauthier ML Beaudry F Vachon P. Intrathecal [6]-gingerol administration alleviates peripherally induced neuropathic pain in male Sprague-Dawley rats. Phytother Res 2013; 27: 1251–1254. 22972597 10.1002/ptr.4837

[172] Oboh G Ademiluyi AO Akinyemi AJ. Inhibition of acetylcholinesterase activities and some pro-oxidant induced lipid peroxidation in rat brain by two varieties of ginger (*Zingiber officinale*). *Experim Toxicol Pathol* 2012; 64: 315–319. 10.1016/j.etp.2010.09.004 20952170

[173] Xue J Li H Deng X, et al. L-Menthone confers antidepressant-like effects in an unpredictable chronic mild stress mouse model via NLRP3 inflammasome-mediated inflammatory cytokines and central neurotransmitters. Pharmacol Biochem Behav 2015; 134: 42–48. 25937574 10.1016/j.pbb.2015.04.014

[174] Chuchawankul S Khorana N Poovorawan Y. Piperine inhibits cytokine production by human peripheral blood mononuclear cells. Genet Mol Res 2012; 11: 617–627. 22535397 10.4238/2012.March.14.5

[175] Ahmad MP Hussain A Siddiqui HH, et al. Effect of methanolic extract of *Asparagus racemosus* Willd. on lipopolysaccharide induced-oxidative stress in rats. Pakistan J Pharmaceut Sci 2015; 28: 509–513. 25730806

[176] Singh GK Garabadu D Muruganandam AV, et al. Antidepressant activity of *Asparagus racemosus* in rodent models. Pharmacol Biochem Behav 2009; 91: 283–290. 18692086 10.1016/j.pbb.2008.07.010

[177] Krishnamurthy S Garabadu D Reddy NR. *Asparagus racemosus* modulates the hypothalamic-pituitary-adrenal axis and brain monoaminergic systems in rats. Nutr Neurosci 2013; 16: 255–261. 23485433 10.1179/1476830513Y.0000000053

[178] Meena J Ojha R Muruganandam AV, et al. *Asparagus racemosus* competitively inhibits *in vitro* the acetylcholine and monoamine metabolizing enzymes. Neurosci Lett 2011; 503: 6–9. 21843599 10.1016/j.neulet.2011.07.051

[179] Ojha R Sahu AN Muruganandam AV, et al. *Asparagus racemosus* enhances memory and protects against amnesia in rodent models. Brain Cognit 2010; 74: 1–9. 20594636 10.1016/j.bandc.2010.05.009

[180] Fatima N Rahman MM Khan MA, et al. A review on *Ipomoea carnea*: Pharmacology, toxicology and phytochemistry. J Complem Integrat Med 2014; 11: 55–62. 10.1515/jcim-2013-0046 24651023

[181] Press JR Shrestha KK Sutton DA. Annotated checklist of the flowering plants of Nepal http://www.efloras.org/flora_page.aspx?flora_id=110 (accessed 3 April 2018).

[182] Baba Ramdev Health Products. Banphool Oil, http://www.ramdevproducts.com/banphool-oil.htm (2018, accessed 1 April 2018).

[183] Kaur M Prakash A Kalia AN. Neuroprotective potential of antioxidant potent fractions from *Convolvulus pluricaulis* Chois. in 3-nitropropionic acid challenged rats. Nutr Neurosci 2016; 19: 70–78. 25896328 10.1179/1476830515Y.0000000022

[184] Agarwa P Sharma B Fatima A, et al. An update on Ayurvedic herb *Convolvulus pluricaulis* Choisy. Asian Pac J Trop Biomed 2014; 4: 245–252. 25182446 10.1016/S2221-1691(14)60240-9 PMC3868798

[185] Sharma K Bhatnagar M Kulkarni SK. Effect of *Convolvulus pluricaulis* Choisy and *Asparagus racemosus* Willd on learning and memory in young and old mice: A comparative evaluation. Indian J Exp Biol 2010; 48: 479–485. 20795365

[186] Heba M Faraz S Banerjee S. Effect of Shankhpushpi on alcohol addiction in mice. Pharmacogn Mag 2017; 13: S148–S153. 28479740 10.4103/0973-1296.203976 PMC5407107

[187] Bihaqi SW Singh AP Tiwari M. Supplementation of *Convolvulus pluricaulis* attenuates scopolamine-induced increased tau and amyloid precursor protein (AbetaPP) expression in rat brain. Indian J Pharmacol 2012; 44: 593–598. 23112420 10.4103/0253-7613.100383 PMC3480791

[188] Malik J Choudhary S Kumar P. Protective effect of *Convolvulus pluricaulis* standardized extract and its fractions against 3-nitropropionic acid-induced neurotoxicity in rats. Pharm Biol 2015; 53: 1448–1457. 25853968 10.3109/13880209.2014.984856

[189] Malik J Karan M Vasisht K. Nootropic, anxiolytic and CNS-depressant studies on different plant sources of shankhpushpi. Pharm Biol 2011; 49: 1234–1242. 21846173 10.3109/13880209.2011.584539

[190] Bihaqi SW Sharma M Singh AP, et al. Neuroprotective role of *Convolvulus pluricaulis* on aluminium induced neurotoxicity in rat brain. J Ethnopharmacol 2009; 124: 409–415. 19505562 10.1016/j.jep.2009.05.038

[191] Flowers of India. Kewda, http://www.flowersofindia.net/catalog/slides/Kewda.html (2018, accessed 28 May 2018).

[192] Rajeswari J Kesavan K Jayakar B. Phytochemical and pharmacological evaluation of prop roots of *Pandanus fascicularis* Lam. Asian Pac J Trop Med 2011; 4: 649–653. 21914545 10.1016/S1995-7645(11)60165-X

[193] Okugawa H Ueda R Matsumoto K, et al. Effect of alpha-santalol and beta-santalol from sandalwood on the central nervous system in mice. Phytomedicine 1995; 2: 119–126. 23196153 10.1016/S0944-7113(11)80056-5

[194] d' Avila Farias M Oliveira PS Dutra FS, et al. Eugenol derivatives as potential anti-oxidants: Is phenolic hydroxyl necessary to obtain an effect? J Pharm Pharmacol 2014; 66: 733–746. 24372555 10.1111/jphp.12197

[195] Halder S Mehta AK Kar R, et al. Clove oil reverses learning and memory deficits in scopolamine-treated mice. Planta Med 2011; 77: 830–834. 21157682 10.1055/s-0030-1250605

[196] Ardjmand A Fathollahi Y Sayyah M, et al. Eugenol depresses synaptic transmission but does not prevent the induction of long-term potentiation in the CA1 region of rat hippocampal slices. Phytomedicine 2006; 13: 146–151. 16428020 10.1016/j.phymed.2004.06.025

[197] Liu BB Luo L Liu XL, et al. Essential oil of *Syzygium aromaticum* reverses the deficits of stress-induced behaviors and hippocampal p-ERK/p-CREB/brain-derived neurotrophic factor expression. Planta Med 2015; 81: 185–192. 25590367 10.1055/s-0034-1396150

[198] Jeon SJ Kim E Lee JS, et al. Maslinic acid ameliorates NMDA receptor blockade-induced schizophrenia-like behaviors in mice. Neuropharmacology 2017; 126: 168–178. 28899728 10.1016/j.neuropharm.2017.09.014

[199] Hartnoll G Moore D Douek D. Near fatal ingestion of oil of cloves. Arch Dis Child 1993; 69: 392–393. 8215554 10.1136/adc.69.3.392 PMC1029532

[200] Lane BW Ellenhorn MJ Hulbert TV, et al. Clove oil ingestion in an infant. Hum Exp Toxicol 1991; 10: 291–294. 1679654 10.1177/096032719101000410

[201] Soubir T. Antioxidant activities of some local bangladeshi fruits (*Artocarpus heterophyllus*, *Annona squamosa*, *Terminalia bellirica*, *Syzygium samarangense*, *Averrhoa carambola* and *Olea europa*). Sheng Wu Gong Cheng Xue Bao 2007; 23: 257–261. 17460898

[202] Chalise JP Acharya K Gurung N, et al. Antioxidant activity and polyphenol content in edible wild fruits from Nepal. Int J Food Sci Nutr 2010; 61: 425–432. 20187713 10.3109/09637481003591590

[203] Tanaka M Kishimoto Y Saita E, et al. *Terminalia bellirica* extract inhibits low-density lipoprotein oxidation and macrophage inflammatory response in vitro. Antioxidants 2016; 5: 20. 10.3390/antiox5020020 PMC4931541 27314393

[204] Jayesh K Helen LR Vysakh A, et al. Ethyl acetate fraction of *Terminalia bellirica* (Gaertn.) Roxb. fruits inhibits proinflammatory mediators via down regulating nuclear factor-kappaB in LPS stimulated Raw 264.7 cells. Biomed Pharmacother 2017; 95: 1654–1660. 28954384 10.1016/j.biopha.2017.09.080

[205] Dhingra D Valecha R. Evaluation of antidepressant-like activity of aqueous and ethanolic extracts of *Terminalia bellirica* Roxb. fruits in mice. Indian J Exp Biol 2007; 45: 610–616. 17821856

[206] Manandhar K Risal A Linde M, et al. The burden of headache disorders in Nepal: Estimates from a population-based survey. J Headache Pain 2015; 17: 3. 26810487 10.1186/s10194-016-0594-0 PMC4726638

[207] Kunwar RM Mahat L Acharya RP, et al. Medicinal plants, traditional medicine, markets and management in far-west Nepal. J Ethnobiol Ethnomed 2013; 9: 24. 23587109 10.1186/1746-4269-9-24 PMC3643841

[208] Kristoffersen ES Lundqvist C. Medication-overuse headache: A review. J Pain Res 2014; 7: 367–378. 25061336 10.2147/JPR.S46071 PMC4079825

[209] Diener HC Dodick D Evers S, et al. Pathophysiology, prevention, and treatment of medication overuse headache. Lancet Neurol 2019; 18: 891–902. 31174999 10.1016/S1474-4422(19)30146-2

[210] Nepalese Ministry of Health and Population. Nepal population report 2011. Kathmandu: Government of Nepal, 2011, pp.1–186.

[211] Risal A Manandhar K Steiner TJ, et al. Estimating prevalence and burden of major disorders of the brain in Nepal: Cultural, geographic, logistic and philosophical issues of methodology. J Headache Pain 2014; 15: 51. 25127664 10.1186/1129-2377-15-51 PMC4149310

